# Emotional Well‐Being Trajectories Before and After Statutory Retirement—Contributions of Social and Health‐Related Factors

**DOI:** 10.1111/sjop.70071

**Published:** 2026-01-14

**Authors:** Emmi‐Susanna Katajapuu, Pauliina Saha, Aapo Hiilamo, Jatta Valkonen, Tea Lallukka

**Affiliations:** ^1^ Department of Public Health University of Helsinki Helsinki Finland; ^2^ Max Planck Institute for Demographic Research Rostock Germany; ^3^ Max Planck – University of Helsinki Center for Social Inequalities in Population Health Helsinki Finland

## Abstract

Aging population in OECD countries and the rising mental disorder burden highlight the need to understand statutory retirement's contribution to emotional well‐being. However, the relationship between statutory retirement and emotional well‐being remains underexplored. Clarifying this relationship can help policymakers enhance pension systems to better support statutory retirees' well‐being. This study examined emotional well‐being trajectories 15 years before and after statutory retirement among 5076 City of Helsinki employees in Finland (81% women; age range 40–60 at Phase 1), and the social and health‐related factors associated with these trajectories. We used prospective cohort data from the Helsinki Health Study (2000–2022) across five phases. Growth Mixture Modeling identified the trajectories, measured by the RAND‐36 emotional well‐being dimension. Multinomial logistic regression with average marginal effects (AMEs) and 95% confidence intervals assessed the associations between social and health‐related factors and these trajectories. A three‐trajectory solution was selected: ‘Stable high’ (85%), ‘Slowly increasing’ (12%), and ‘Fast increasing, then fast decreasing’ (3%) emotional well‐being. Mentally very strenuous work, binge drinking, smoking, frequent sleep problems, and mental disorder diagnoses before retirement were associated with lower predicted probabilities of the ‘Stable high’ trajectory and higher predicted probabilities of the ‘Slowly increasing’ trajectory. Mentally very strenuous work and mental disorder diagnoses were linked to a higher predicted probability of the ‘Fast increasing, then fast decreasing’ trajectory. Most participants maintained high emotional well‐being throughout the statutory retirement transition. A smaller group of individuals experienced lower emotional well‐being before statutory retirement and a gradual improvement after, or saw an increase until retirement, followed by a rapid decline. Mentally very strenuous work, binge drinking, smoking, frequent sleep problems, and mental disorder diagnoses before retirement were associated with poorer emotional well‐being trajectories. With targeted interventions we could explore whether a change in these factors could enhance emotional well‐being across retirement.

## Keypoints

- We explored emotional well‐being trajectories before and after statutory retirement transition.
- Most retirees maintained high emotional well‐being over the 15 year follow‐up.
- A small group experienced lower emotional well‐being before retirement and a gradual improvement after; another saw an increase until retirement, followed by a rapid decline.
- Pre‐retirement work strenuousness and unhealthy behaviors were associated with poorer emotional well‐being trajectories.

## 1 Introduction

Population aging is a global trend, with the proportion of individuals aged over 65 in Organization for Economic Co‐operation and Development (OECD) countries nearly doubling from under 9% in 1960 to 17% in 2015, and is projected to reach 28% by 2050 (OECD 2023a). The burden of mental disorders is also increasing globally, including Finland, which has the highest estimated incidence of mental disorders in the EU across its population (OECD 2019, 2023a; Wu et al. 2023). According to the World Health Organization, 14% of people over the age of 60 live with a mental disorder globally, and older adults may be at an increased risk of developing mental health problems such as depression and anxiety (World Health Organization 2023). Given this, understanding the emotional well‐being of older adults has become increasingly essential to effectively support the well‐being of this growing share of the population.

One common life event that is often linked to emotional well‐being is statutory retirement (van der Heide et al. 2013), which is the most common pathway out of the workforce for older individuals (Eurostat 2020). In response to an aging population, many OECD countries have raised their retirement ages and modified statutory pension systems (OECD 2023b). For example, Finland reformed its pension system in 2017, adjusting retirement ages to base on life expectancy (Finnish Centre for Pensions 2024). Evidence on the link between statutory retirement and emotional well‐being could help policymakers balance economic demands with the social and health needs of retirees, promoting a high quality of life in older age. For example, if statutory retirement contributes positively to emotional well‐being but careers are getting longer, improving working conditions throughout careers to support emotional well‐being could help make longer careers sustainable for both individuals and society.

Retirement has long been acknowledged as one of the most significant life events requiring adjustment (Ekerdt 1987). Consequently, a large body of research has examined the relationship between statutory retirement and emotional well‐being. This research generally indicates that the statutory retirement transition is associated with improvements in emotional well‐being (Fleischmann et al. 2020; Mänty et al. 2018; Min et al. 2023; Stenholm et al. 2020; van der Heide et al. 2013; Vo and Phu‐Duyen 2023; Westerlund et al. 2010).

A growing body of research has also utilized person‐oriented approaches to explore latent groups or trajectories of well‐being over the retirement transitions. Person‐oriented approaches can identify nuanced emotional well‐being trajectories that variable‐oriented approaches looking at grand means may overlook. For example, Min et al. (2023) identified three latent groups of mental health severity—mild, moderate, and severe—among older adults aged 64–75 at baseline. Their findings revealed that individuals were more likely to shift into less severe categories as they aged. The potential mechanisms through which retirement contributes to improved emotional well‐being are numerous, including elimination of work‐related stressors (Eibich 2015; Pinquart 2002) and retirees having more time for leisure and sleep (Dave et al. 2008; Eibich 2015).

There is also evidence of emotional well‐being either staying stable or deteriorating after retirement (Åhlin et al. 2020; Gruszczyńska et al. 2019; Heller‐Sahlgren 2017; Horner and Cullen 2016; Laaksonen et al. 2012; Segel‐Karpas et al. 2018; Shiba et al. 2017). For instance, Segel‐Karpas et al. (2018) found no significant direct change in depressive symptoms following retirement, but that loneliness significantly increased depressive symptoms, and the effect was stronger among retirees than the employed.

Many pre‐retirement social and health‐related factors can contribute to the link between retirement transitions and mental well‐being trajectories. For example, Fleischmann et al. (2020) found that individuals who experienced favorable working conditions before statutory retirement tended to have better mental health after retiring. They also discovered that those who were not on antidepressants had significantly more improvement in their mental health during statutory retirement transition than those who were. Similarly, Min et al. (2023) found in their latent group analysis of older adults that factors like lower education, smoking, and poorer sleep were more prevalent in groups with more severe mental health symptoms. Other social and health‐related factors relating to better emotional well‐being outcomes at older age are, for instance, high parental educational level (Sutin et al. 2018), being married (Dave et al. 2008), high levels of physical activity (Kim et al. 2017), no binge‐drinking behavior (Lallukka et al. 2020), high consumption of fruit and vegetables (Głąbska et al. 2020), normal body mass index (BMI) (Stenholm et al. 2017), and no sleep problems (Lallukka et al. 2020). Identifying different social and health‐related factors contributing to emotional well‐being trajectories could help us to plan tailored interventions for those at risk for worse well‐being during the retirement transition.

However, one challenge in interpreting these previous findings is the lack of distinction between statutory retirement and other types of retirement, such as disability retirement, which may yield different health outcomes (see e.g., Jokela et al. 2010; Laaksonen et al. 2012). This distinction is critical given that statutory retirement is the most common form, warranting more focused investigation. Moreover, much of the existing research has been cross‐sectional or has adopted only short‐term follow‐ups. For example, Gruszczyńska et al. (2019) identified four distinct trajectories of each of the following dimensions of well‐being: satisfaction with life, depression, and subjective health following statutory retirement. None of the trajectories indicated improvement in health, but their study only spanned 1 year after retirement, thus not capturing longer‐term patterns. In contrast, Westerlund et al. (2010) found that both mental fatigue and depression significantly decreased during the year leading up to retirement and the year following it, with both conditions stabilizing at lower levels than before retirement, observed in a total of 7 years' follow‐up.

This study seeks to address these gaps present in previous research by exploring emotional well‐being trajectories among former Finnish municipal employees, utilizing a person‐oriented approach and a follow‐up period of 15 years before and 15 years after retirement, and considering a broad range of social and health‐related factors. The research questions are:

1. What kinds of emotional well‐being trajectories can be observed before and after statutory retirement among former Finnish municipal employees?
2. How are social‐ and health‐related factors prior to retirement transition associated with the identified emotional well‐being trajectories?

## 2 Methods

### 2.1 Data

We used data from the Helsinki Health Study, which is an ongoing longitudinal study that has gathered information about health and its determinants of employees of the City of Helsinki, Finland, since 2000 (Lahelma et al. 2013). Phase 1 data were derived from surveys conducted between 2000 and 2002 among employees of age 40, 45, 50, 55 or 60 (*N* = 8960; response rate 67%). Follow‐up surveys thus far have been conducted in 2007 (Phase 2, *N* = 7332; response rate 83%), 2012 (Phase 3, *N* = 6814; response rate 79%), 2017 (Phase 4, *N* = 6832; response rate 82%) and 2022 (Phase 5, *N* = 5944; response rate 75%) among those who responded at Phase 1. The follow‐up surveys were sent to the participants even if they had already retired or were currently working somewhere else than the City of Helsinki. Retirement was assessed by asking the type of possible pension and its starting year and month at each follow‐up phase. All participants were working at Phase 1 and 86% of them had retired by Phase 5. Participants who statutorily retired during the follow‐up period were included in this study. Timing of statutory retirement was determined from the latest phase where a participant reported it; if there was inconsistent or missing information about the timing of retirement, the participant was excluded (*N* = 177). Only participants with data on emotional well‐being from at least three survey phases were included (95%) to more reliably model development in emotional well‐being trajectories. The final sample consisted of 5076 participants of whom 81% were women, reflecting the target population (Lahelma et al. 2013). The selection criteria of the sample are displayed in Figure S1.

Strict General Data Protection Regulation (GDPR) laws prevent from providing the data publicly available. More details are available from the Helsinki Health Study data protection statement: www.helsinki.fi/hhs_dataprotectionstatement. The Helsinki Health Study protocol was approved by the Ethics Committee of the Faculty of Medicine, University of Helsinki, and the City of Helsinki health and personnel authorities.

### 2.2 Measures

#### 2.2.1 Emotional Well‐Being

Emotional well‐being was measured with the RAND‐36 questionnaire, which is a validated measure of health‐related quality of life (Hays et al. 1993). The measure is comprised of eight dimensions: emotional well‐being, general health, physical health, limitations due to physical health issues, limitations due to emotional issues, social functioning, pain, and fatigue/energy. We used the dimension of emotional well‐being, which was measured with five questions assessing well‐being during the past 4 weeks with six response alternatives ranging from “all of the time” to “none of the time”. The questions were the following: “Have you been very nervous?”, “Have you felt so down in the dumps that nothing could cheer you up?”, “Have you felt calm and peaceful?”, “Have you felt downhearted and blue?”, and “Have you been a happy person?”. According to the RAND scoring guidelines, responses to each item were transformed to 0, 20, 40, 60, 80 or 100. Next, a mean score was calculated resulting in a final score ranging from 0 to 100, with higher scores indicating better health functioning (Hays et al. 1993). We reverse scored the third and fifth items so that a total score could be computed. The follow‐up period of emotional well‐being from the first point of measurement ranged among the study participants from 10 to 22 years with an average follow‐up of 21 years. The follow‐up period following retirement varied from 0 to 22 years with an average of 9 years. The RAND‐36 has shown to have good psychometric properties, including good criterion and convergent validity as well as good reliability (Cronbach's alphas more than 0.80 for all dimensions, Ohlsson‐Nevo et al. 2021; Andersen et al. 2022). The emotional well‐being score of the RAND‐36 is identical to the Mental Health Index (MHI‐5) dimension of the SF‐36 questionnaire (Hays et al. 1993), which is often also used in literature as a measure of emotional well‐being.

#### 2.2.2 Social and Health‐Related Factors

All social and health‐related factors were selected based on prior research relating to emotional well‐being with retirement (Åhlin et al. 2020; Fleischmann et al. 2020; Kontturi et al. 2024; Lahdenperä et al. 2021; Lahti, Knop, et al. 2023; Lahti, Salmela, et al. 2023; Lallukka et al. 2018; Mänty et al. 2018; Mauramo et al. 2023). Looking at the exposures prior to retirement allowed us to assess which factors should be given special consideration to enhance well‐being already before retirement.

##### 2.2.2.1 Social Factors

Social factors included sociodemographic and socioeconomic factors as well as working conditions. The following social factors were from Phase 1: participant's age (continuous), gender (women and men), parental education level, marital status (married/cohabiting and others; Holstila et al. 2017), and participant's education level. Physical and mental strenuousness of work were derived from the phase before the participant's retirement.

Parental and participant's own education levels were classified as higher education (university degree or more), intermediate education (matriculation or equivalent), and basic education (primary or secondary school or less) (Mauramo et al. 2023). Parental education level was based on maternal and paternal educational levels, of which the higher one was chosen (Mauramo et al. 2023). Physical and mental strenuousness of work were evaluated with single questions asking how strenuous participants considered their work physically and mentally. The answering options were “very light”, “rather light”, “rather heavy” and “very heavy”. We classified the physical and mental strenuousness of work into three categories based on previous research (Lahti et al. 2021). Physical strenuousness was classified into physically non‐strenuous (very light), intermediately strenuous (rather light), and strenuous (rather strenuous or very strenuous) work. Mental strenuousness was classified into mentally non‐strenuous (very light or rather light), intermediately strenuous (rather strenuous), and strenuous (very strenuous).

##### 2.2.2.2 Health‐Related Factors

Health‐related factors included lifestyle factors and mental health problems, including leisure‐time physical activity (LTPA), binge drinking, smoking, fruit and vegetable consumption (F&V), BMI, sleep problems, and mental disorder diagnoses. All health‐related factors were determined from the phase before each participant's retirement.

Metabolic equivalent of task (MET) hours were computed based on the participants' report of their average weekly amount of LTPA, including commuting exercise, in hours within the past 12 months. Data were collected in four categories of increasing intensity, including walking, brisk walking, jogging, and running, as well as their equivalent activities. We then classified LTPA into three categories: vigorous activity (14 MET hours or more per week of activity in the highest two intensity categories), moderate activity (14 MET hours or more per week of activity in the lowest two intensity categories), and inactivity (less than 14 MET hours per week) (Holstila et al. 2017).

Binge drinking was evaluated through a single question asking how often the participant ingests six or more units of alcohol in a single instance. The question had six response alternatives ranging from “never” to “daily or almost daily”, and we classified answers into having no binge drinking behavior (once a month or less) and having binge drinking behavior (once a week or more) (Saha et al. 2025). Smoking was assessed with a single question inquiring whether the participant smoked currently either cigarettes, cigars, or pipe tobacco regularly, with answer options “yes” and “no”, and thus, we classified answers into “current non‐smoker” and “current smoker” (Suur‐Uski et al. 2024). F&V was assessed by asking the participant how often they consumed fresh vegetables or fruit and berries in the past 4 weeks, as derived from a 20‐item food frequency questionnaire. Response options ranged from “not at all” to “twice a day or more often”, and we classified answers into daily consumption (both fruit and vegetables consumed daily) and non‐daily consumption (fruit and vegetables less than daily) (Salmela et al. 2021).

BMI was calculated from participants' self‐reported height and weight (kg/m^2^) and divided into three categories: normal weight (BMI < 25.0 kg/m^2^), overweight (25.0 ≤ BMI < 30.0 kg/m^2^) and obesity (BMI ≥ 30.0 kg/m^2^) (World Health Organization 2010). The proportion of those often considered with underweight (BMI < 18.5 kg/m^2^) was too small (0.8%) for statistical analyses and they were thus included in the reference category of normal weight. Self‐reported sleep problems were assessed through a four‐item version of the Jenkins questionnaire (Jenkins et al. 1988). The questionnaire assesses sleep problems during the past four weeks with six answer options ranging from “never” to “on 22–28 nights”. The dimensions of sleep problems assessed are difficulty falling asleep, frequent awakenings during the night, trouble remaining asleep, and subjective feelings of fatigue and sleepiness despite receiving a typical night's rest (Shahid et al. 2012). We classified sleep problems into three categories, including having no problems (never in each dimension), having occasional problems (any symptoms during 14 nights per 4 weeks or less), and having frequent problems (any symptoms during more than 14 nights per 4 weeks) (Salmela et al. 2021). Mental disorder diagnoses were assessed with a single question asking whether the participant had ever before received a diagnosis of depression, anxiety disorder, or any other mental disorder from a doctor before, with answer options “yes” and “no”.

### 2.3 Missing Data

For factors derived from the phase prior to participant's retirement, if a participant did not take part in the phase immediately before their retirement, information was derived from the phase preceding it. For most factors, information from the phase immediately before retirement was available for 95%–97% of participants. However, for physical and mental workload strenuousness, only 36% of participants had data from this phase.

Missing information on most of the social and health‐related factors was minimal (0%–3%) in which case the missing cases were included in the most ‘favorable’ category of the factor as a conservative way to handle missing data and as per previous research (Etholén et al. 2022). Thus, we placed missing information on parental educational level (3%) to the category ‘higher education’, marital status (1%) to ‘married/cohabiting’, participant's own educational level (1%) to ‘higher education’, LTPA (2%) to ‘vigorous’, binge drinking (3%) to ‘no binge drinking’, smoking (2%) to ‘no smoking’, F&V consumption (2%) to ‘daily consumption’, BMI (2%) to ‘normal weight’, and sleep problems (2%) to ‘never’. Missing data on mental disorder diagnoses (10%) and physical and mental strenuousness of work (14% and 15%, respectively) were relatively high. This could possibly relate to not bothering responding “no” to each item in the long list of diagnoses or preferring not to report a diagnosis or workload strenuousness. Handling these missing data as their own category helped tackle this.

### 2.4 Statistical Analyses

Descriptive statistics for the emotional well‐being scores and the social and health‐related factors were computed, after which Chi‐squared tests were computed for the social and health‐related factors. To ensure that there was no multicollinearity among the social and health‐related factors, Cramer's V correlation matrix was checked.

We then utilized Growth Mixture Modeling (GMM) to determine trajectories of emotional well‐being, enabling the identification of several unobserved sub‐populations and the analysis of variations in their changes. GMM identifies latent subgroups within a population that differ in their initial status and developmental trajectories over time. Rather than assuming a single homogeneous population, GMM allows for the estimation of multiple distinct classes, each characterized by its own growth parameters. In interpreting the identified classes, it is essential to acknowledge that they are derived as non‐parametric representations of heterogeneity in individual trajectories. Rather than reflecting inherently meaningful or substantively distinct typologies, they serve as statistical approximations of variation across individuals over time (Frankfurt et al. 2016; Herle et al. 2020). GMM uses all available repeated‐measures data per individual under the assumption that data are missing at random (Proust‐Lima et al. 2017).

For the trajectories, the x‐axis represents the timing of statutory retirement with zero point representing the year of retirement. As such, it shows the development of emotional well‐being as a function of retirement, showing development before and after retirement. Models including up to six trajectory groups were estimated. We used Bayesian Information Criterion (BIC), Akaike Information Criterion (AIC), the average posterior probabilities (APP) of group membership (over 0.70) (Nagin and Odgers 2010) and the interpretability of the trajectories as the criteria determining the most fitting model. These criteria were chosen because they have been commonly used in previous studies (see, for example, Etholén et al. 2024; Herle et al. 2020; Senay et al. 2021). Participants were assigned to the trajectory group in which they had the highest posterior probability of belonging. The models were fitted with and without gender interaction and the results remained similar in both cases, and thus we report the simpler model without gender interaction. The trajectory groups ‘Stable high’ and ‘Slowly increasing’ were most similar with and without gender interactions. The change in the trajectory group ‘Fast increasing, then fast decreasing’ was slightly steeper prior to retirement and years after retirement without gender interaction. The model fit statistics are provided in Table S1, and they can be observed as plots in Figures S2 and S3.

After identifying the emotional well‐being trajectories, we cross‐tabulated the social and health‐related factors with the selected trajectory groups. Then, we performed multinomial logistic regression models to examine the associations between pre‐retirement predictors and the trajectory group membership. Model 1 was adjusted for age and gender. We then adjusted model 1 further in model 2 for other social factors (parental education, marital status, participant's own education, and physical and mental strenuousness of work) (model 2). Lastly, we adjusted model 1 further in model 3 for health‐related factors (LTPA, alcohol consumption, smoking, F&V, BMI, sleep problems, and mental disorder diagnoses) (model 3). Using these three models allows us to incrementally account for different sets of factors, thereby assessing the robustness and relative impact of these on the outcome. To avoid rounding issues in the regression analyses, age was coded as a continuous variable representing decades (i.e., age divided by 10). The results of the regression analyses are presented as average marginal effects (AME) with 95% confidence intervals (CI). AMEs represent the differences in predicted probabilities of an outcome for different levels of the predictor variable within each trajectory group (Leeper 2017).

All analyses were performed using R version 4.3.3. For the trajectory analyses, we used the package “LCMM” (Proust‐Lima et al. 2017) and for the multinomial logistic regression analyses we used the package “nnet” (Liang et al. 2020).

## 3 Results

The descriptive statistics are displayed in Tables S2–S5. The mean age of participants at Phase 1 was 52 (SD 5), at the phase prior to retirement was 61 (SD 3), and at retirement was 65 (SD 2) years. Over the follow‐up, emotional well‐being was higher among those who retired compared to those who had not yet retired at each phase (Table S1). Among retirees, men typically reported higher emotional well‐being than women. Most participants did not report binge‐drinking (91%) or smoking (86%) at the phase before their retirement. Only 11% of participants reported not experiencing any sleep problems in the 4 weeks preceding the phase prior to their retirement. The correlation matrix revealed no multicollinearity between any variables (see Figure S4).

**FIGURE 1:**
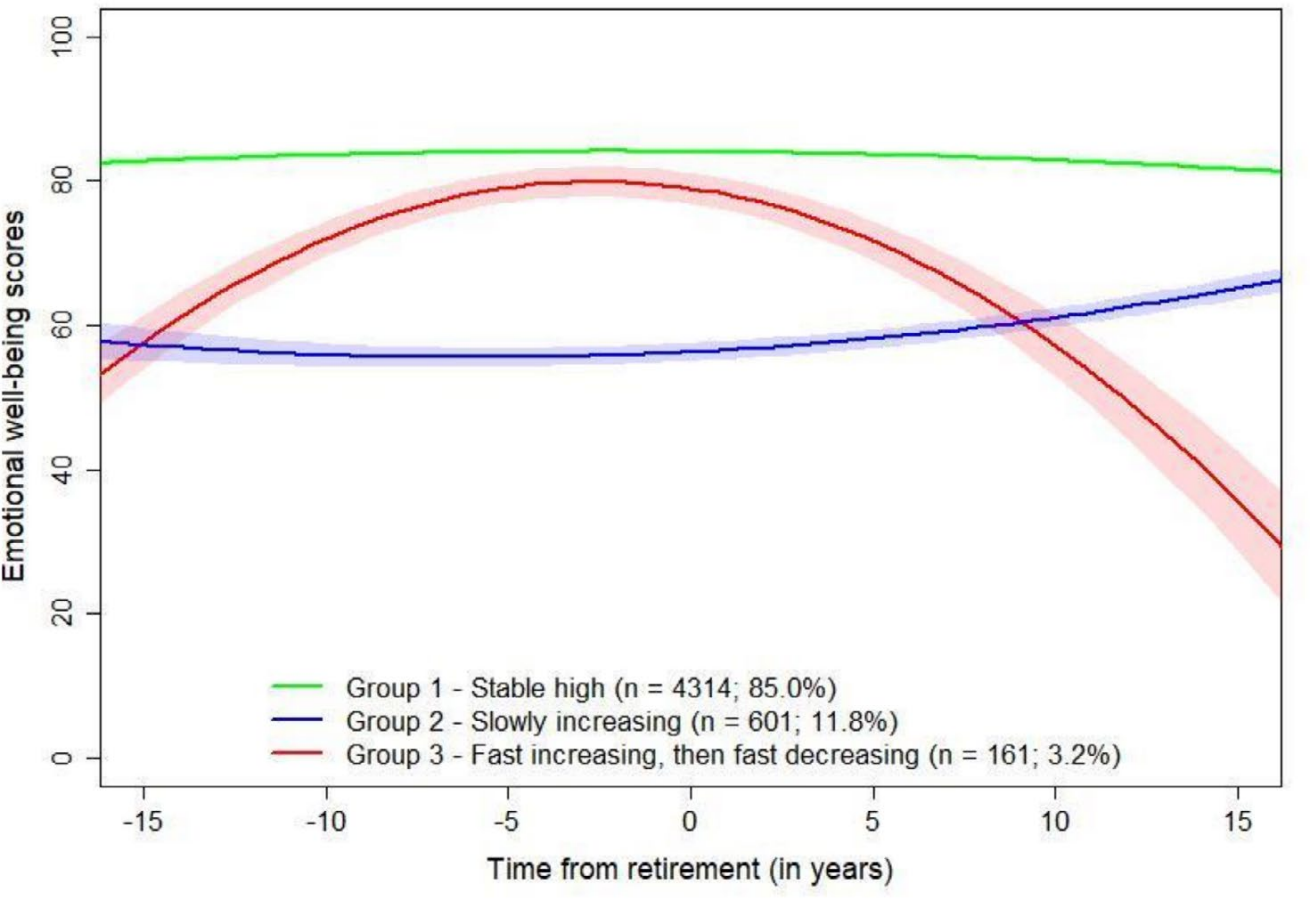
Emotional well‐being trajectories of statutory retirees with group means and fitted lines with 95% confidence intervals, identified by growth mixture modeling. Number of participants in each group and the prevalence of group sizes are shown. X‐axis shows years before and after retirement with 0 indicating the retirement year. The Helsinki Health Study 2000–2022 (*N* = 5076, 81% women).

A trajectory model with three emotional well‐being trajectories was selected (see Figure 1 and Table S1). This model demonstrated the lowest BIC and AIC values, adequate posterior probabilities, and did not yield trajectory groups without assigned participants, unlike models with more classes. We only report the solutions up to four classes as the four‐class solution already showed signs of overfitting (two groups without allocated participants), and models with additional classes further worsened model fit (higher BIC/AIC values) without yielding interpretable trajectories.

The first trajectory group, ‘Stable high’, included 85% (*N* = 4314) of the participants; the second group, ‘Slowly increasing’, included 12% (*N* = 601) of the participants; and the third group, ‘Fast increasing, then fast decreasing’, included 3% (*N* = 161) of the participants. The ‘Stable high’ trajectory group had relatively high scores in emotional well‐being throughout the follow‐up. The ‘Slowly increasing’ trajectory group started from moderately high emotional well‐being scores which slowly started to increase after retirement. The ‘Fast increasing, then fast decreasing’ trajectory group had steadily and steeply increasing scores before retirement which stabilized at relatively high around retirement; following retirement, the scores started to steeply decrease. Time from retirement was truncated at years −15 and 15 as there were not many emotional well‐being score values per trajectory group past these points (see Figure S5). The underlying estimates for each trajectory group are available in Figures S6–S8.

Having a mentally non‐strenuous job was more typical in the ‘Stable high’ trajectory group (26%) than the ‘Slowly increasing’ (15%) or the ‘Fast increasing, then fast decreasing’ (11%) trajectory groups (see Table S5). Binge‐drinking and smoking were more typical in the ‘Slowly increasing’ (binge‐drinking 16%; smoking 24%) and the ‘Fast increasing, then fast decreasing’ (binge‐drinking 19%; smoking 22%) trajectory groups than the ‘Stable high’ trajectory group (binge‐drinking 8%; smoking 12%). Consuming F&V daily was more common in the ‘Stable high’ trajectory group (59%) than in the other two trajectory groups (‘Slowly increasing’ 45%; ‘Fast increasing, then fast decreasing’ 44%). A bigger proportion of participants reported occasional sleep problems in the ‘Stable high’ trajectory group (67%) than the other groups (‘Slowly increasing’ 45%; ‘Fast increasing, then fast decreasing’ 53%). Those in the ‘Slowly increasing’ trajectory group reported the most frequent sleep problems (52%), compared to the other groups (‘Stable high’ 22%; ‘Fast increasing, then fast decreasing’ 38%). Having had a mental disorder diagnosis was less common in the ‘Stable high’ trajectory group (12%) than in the other two trajectory groups (‘Slowly increasing’ 38%; ‘Fast increasing, then fast decreasing’ 39%).

**FIGURE 2:**
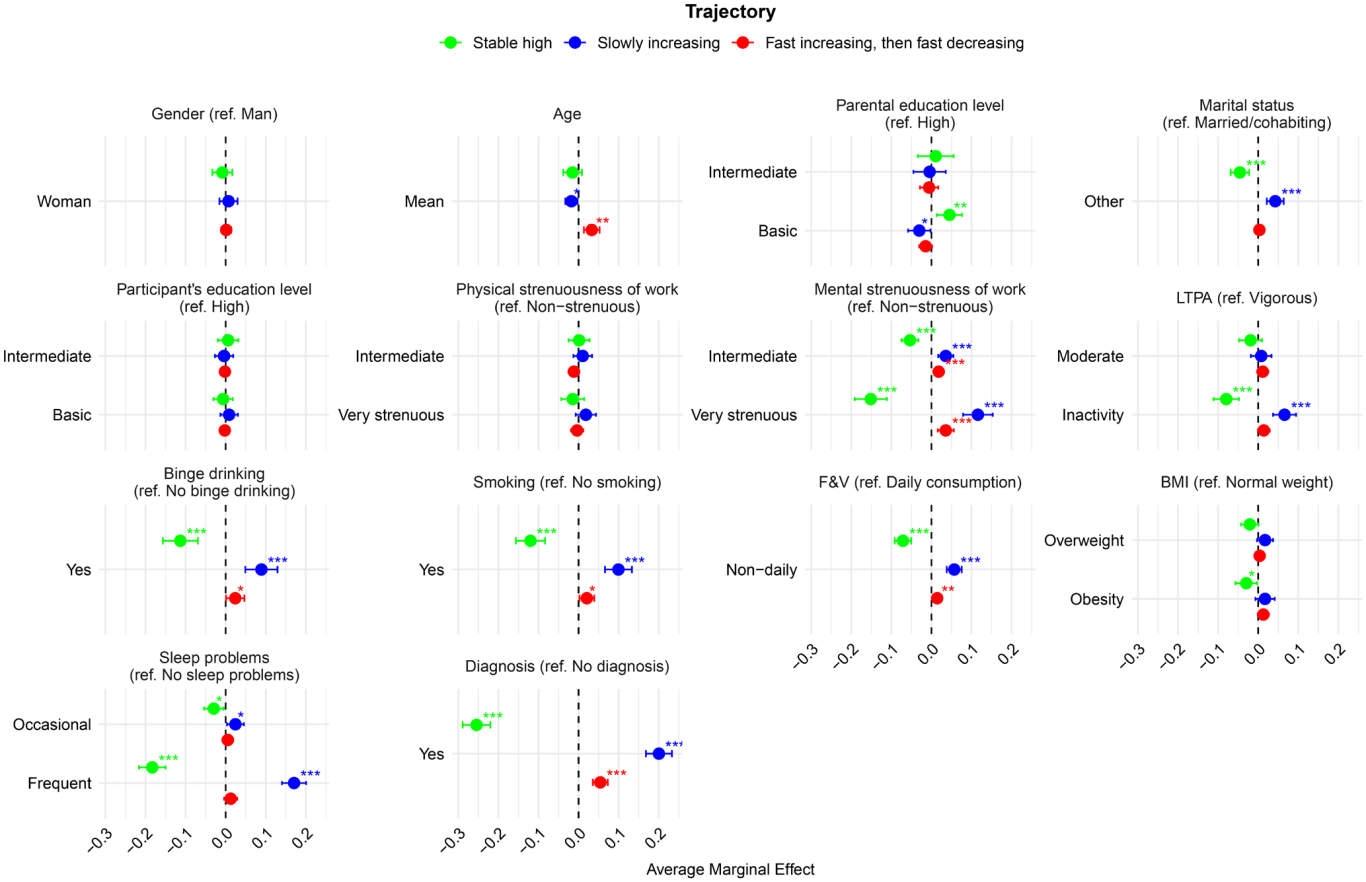
Multinomial regression results of model 1 as average marginal effects (AME) and their 95% confidence intervals for the statutory retirees' emotional well‐being trajectories. The Helsinki Health Study 2000–2022 (*N* = 5076, 81% Women). BMI, body mass index; F&V, fruit and vegetable consumption; LTPA, leisure‐time physical activity. Age represented in increments of 10 years. *p ≤ 0.1; **p ≤ 0.05; ***p ≤ 0.01.

The following results are from model 1 (see Figure 2; see also Table S6 and Figure S9). Individuals with mentally very strenuous jobs were found to have a 15 percentage points (CI 11–19) lower predicted probability of belonging to the ‘Stable high’ trajectory group compared to those in non‐strenuous jobs. In contrast, those with mentally very strenuous jobs had a 12 percentage points (CI 8–15) higher predicted probability of falling into the ‘Slowly increasing’ trajectory and a 4 percentage points (CI 2–6) higher predicted probability of belonging to the ‘Fast increasing, then fast decreasing’ group.

Binge drinking and smoking were similarly associated with lower emotional well‐being over the follow‐up: those who engaged in binge drinking had an 11 percentage points (CI 7–16) lower predicted probability of belonging to the ‘Stable high’ group. Moreover, those with binge‐drinking behavior had a 9 percentage points (CI 5–13) higher predicted probability of falling into the ‘Slowly increasing’ trajectory. Smokers had a 12 percentage points (CI 8–16) lower predicted probability of belonging to the ‘Stable high’ trajectory, with a corresponding 10 percentage points (CI 7–13) higher predicted probability of belonging to the ‘Slowly increasing’ group.

Those with frequent sleep problems had an 18 percentage points (CI 15–22) lower predicted probability of belonging to the ‘Stable high’ trajectory, while having a 17 percentage points (CI 14–20) higher predicted probability of falling into the ‘Slowly increasing’ group. Finally, individuals with a mental disorder diagnosis had a lower predicted probability (26 percentage points, CI 22–29) of belonging to the ‘Stable high’ trajectory. In contrast, they had a 20 percentage points (CI 17–23) higher predicted probability of belonging to the ‘Slowly increasing’ trajectory and a 5 percentage points (CI 4–7) higher probability of the ‘Fast increasing, then fast decreasing’ trajectory.

All these associations remained similar in magnitude in model 2, with a difference in result remaining within a margin of 3 percentage points for each social and health‐related factor (Tables S6 and S7; Figures S10 and S11). In model 3, most of the associations remained, albeit attenuated in magnitude (Tables S6 and S7; Figures S12 and S13).

Additional analyses were conducted in which missing data on all exposure variables were excluded rather than assigned to the most favorable category (data not shown). These analyses demonstrated that all AME estimates were similar to the main analyses.

## 4 Discussion

### 4.1 Main Findings

This study sought to examine how the emotional well‐being of former Finnish municipal employees developed 15 years before and 15 years after statutory retirement. The main finding was that most of the participants were assigned to the trajectory group ‘Stable high’ where emotional well‐being remained high throughout statutory retirement and retirement did not have a notable effect on the trajectory. Additionally, a smaller group of individuals experienced a gradual improvement in emotional well‐being after statutory retirement, or saw an increase until retirement, followed by a rapid decline.

The second main finding was that engaging in unfavorable social and health‐related factors might contribute to lower levels of emotional well‐being before statutory retirement. After retirement, the emotional well‐being of those engaging in these factors might slowly start increasing or start declining fast.

### 4.2 Interpretation

Most of the participants assigned to the trajectory group where emotional well‐being remained high throughout statutory retirement are in line with previous research that has utilized person‐oriented analyses and found similar results in statutory retiree samples (Åhlin et al. 2020; Gruszczyńska et al. 2019). Our study supports the notion that person‐oriented approaches can identify nuanced emotional well‐being trajectories that variable‐oriented approaches may have overlooked in the past, and that most individuals enjoy stably good emotional well‐being throughout the statutory retirement transition.

The ‘Slowly increasing’ trajectory group is in line with former studies which imply that statutory retirement contributes to improved emotional well‐being (Fleischmann et al. 2020; Jokela et al. 2010; Mein et al. 2003; van der Heide et al. 2013). However, we also identified a trajectory group that had the second‐highest emotional well‐being scores at retirement, but 10 years after retirement, their well‐being levels had fallen below those of the ‘Slowly increasing’ group, which had the lowest scores at retirement. As a relatively small number of participants (3%) were assigned to this trajectory group, the results concerning this trajectory group should be interpreted cautiously, as estimates may be unstable, sensitive to model specifications, and limited in statistical power, thus potentially reducing our ability to detect true associations. However, it aligns with previous research showing that retirement has a negative association with emotional well‐being (Heller‐Sahlgren 2017; Segel‐Karpas et al. 2018; Shiba et al. 2017). The person‐oriented approach may contribute to these nuanced findings of emotional well‐being trajectories. This finding suggests that emotional well‐being at retirement does not indicate long‐term well‐being. Raising awareness of these risks can help individuals better prepare for retirement, with resources such as counseling and guidance from employers or healthcare professionals to support their emotional well‐being during the transition.

Looking at the exposures prior to retirement allowed for us to assess which factors should be given special consideration to enhance well‐being already before retirement. As unfavorable work and health conditions were associated with a lower probability of belonging to the most favorable trajectory group, to ensure the best possible emotional well‐being outcome, it may be beneficial to address these negative factors proactively. This also suggests that these factors may serve as key conditions and behaviors contributing to variations in the development of emotional well‐being over time, since they are linked to a lower probability of belonging to the only trajectory group characterized by consistent emotional well‐being throughout statutory retirement.

However, having had a mentally very strenuous job and mental disorder diagnoses were associated with both the ‘Slowly increasing’ trajectory group and the ‘Fast increasing, then fast decreasing’ trajectory group. It could be that, for instance, mental disorder diagnoses could possibly contribute to a person's emotional well‐being differently depending on the level of resilience. For example, Nalin and Franca (2015) found that resilience predicted improved subjective well‐being in retirement so that those who had greater resilience had also greater well‐being. Perhaps those with higher resilience were able to enjoy the benefits brought on by retirement and thus their emotional well‐being started improving despite their mental disorder diagnoses, while for those with lower resilience emotional well‐being started decreasing. Another important consideration is that mental health diagnoses were assessed based on whether an individual had ever received a diagnosis in their lifetime. For example, a diagnosis acquired in adolescence may relate differently to emotional well‐being in older adulthood than one given in adulthood. Future research should examine how resilience or the timing of a diagnosis contributes to the emotional well‐being trajectories.

Binge drinking, smoking, and sleep problems were associated with the ‘Slowly increasing’ trajectory group but not with the ‘Fast increasing, then fast decreasing’ trajectory group. This may be surprising, considering that these factors relate to improving but not deteriorating emotional well‐being after retirement. It could be that the size of the ‘Fast increasing, then fast decreasing’ group is too small to detect all relevant factors associated with it. Another possibility is that individuals change their behavior during statutory retirement, which contributes to emotional well‐being. Since this study prioritized exposures prior to statutory retirement, changes in unfavorable behaviors after retirement were not considered. Future research should assess how such changes might contribute to what emotional well‐being trajectory one is assigned to.

Accordingly, the trajectories may reflect different retiree experiences. Robinson et al. (2011) found in their qualitative interview study that some retirees feel liberated and experience improved well‐being, while others feel a sense of loss and decline. Considering that job stress increases smoking intensity and alcohol consumption (Azagba and Sharaf 2011), and it has a consistently negative association with sleep quality throughout studies (Mao et al. 2023), perhaps the ‘Slowly increasing’ group might represent those with stressful jobs, leading to issues like sleep problems, smoking, and binge drinking. As retirement reduces job stress, their emotional well‐being improves, and they may be more likely to reduce unhealthy behaviors like smoking. For example, statutory retirement has been shown to be a common point after which smokers reduce or quit smoking (Kesavayuth et al. 2018; Pulakka et al. 2018) and sleep problems might alleviate (Vahtera et al. 2009).

In contrast, the ‘Fast increasing, then fast decreasing’ group may include those with mentally strenuous jobs who found purpose and identity in their work, boosting well‐being until retirement (Bordia et al. 2020; Butzer and Kuiper 2006; Jolles et al. 2023; McIntyre et al. 2014; Pinquart 2002). After retirement, the sudden loss of work‐related identity may be linked to a decline in well‐being, as noted by Robinson et al. (2011). Future research should examine how change in social and health‐related factors during statutory retirement relate to emotional well‐being.

Verhaeghen and Hertzog (2014) summarize that as people age, their physiological emotional responses become less intense, which makes regulating emotions easier. Because older adults perceive time as more limited, they are more motivated to manage their emotions and willingly allocate more cognitive resources to do so. Over time, they tend to rely on emotion regulation strategies that are cognitively efficient and become more skilled at using them through practice. It is also noted that older adults tend to prioritize positive over negative information more than younger individuals, and they actively steer clear of situations that might lead to unpleasant emotional experiences (Kryla‐Lighthall and Mather 2009). Then, why is it possible to find a trajectory group such as ‘Fast increasing, then fast decreasing’? There may be unexamined factors associated with different emotional well‐being trajectories. For example, as Segel‐Karpas et al. (2018) found in their study, loneliness may be linked to whether well‐being increases or decreases during retirement, as lonely individuals may experience challenges with losing work connections, while others engage more with loved ones. Also, according to Henry et al. (2023), social frailty notably predicts greater demoralization, social anxiety, and poorer life satisfaction. Future research should explore additional social and health‐related factors, such as social support, in relation to post‐retirement well‐being.

We should strive to identify and support individuals at risk of emotional well‐being decline after statutory retirement. For example, resilience‐building interventions have shown effectiveness in improving emotional well‐being (Joyce et al. 2018; Sayed et al. 2024). In addition, interventions that encourage individuals to reflect positively on their past, stay engaged in the present, and plan meaningfully for the future may help strengthen emotional and health resources during the transition to retirement (Earl and Burbury 2019). Digital solutions such as virtual or online coaching programs could further support this process by helping older workers and retirees clarify their needs and adopt healthier lifestyles as they approach and adjust to retirement (Santini et al. 2021, 2023). Targeted pre‐retirement interventions could help promote healthy lifestyles and positive emotional well‐being outcomes of those in the ‘Fast increasing, then fast decreasing’ trajectory group. Future research should focus on developing and testing such interventions, including scalable and user‐friendly digital health options, which are increasingly accessible and effective (Kasoju et al. 2023).

### 4.3 Limitations and Strengths

We acknowledge limitations of the current study. Although our study considered several social‐ and health‐related factors, it still left several without attention, such as the contribution of resilience and level of social support, as they were not available in our data. In addition, our sample consisted mostly of women, which reflected the target population, but as the proportion of men was low, gender‐stratified analyses were not possible. Notably, among participants with emotional well‐being data from all five phases, gender interaction revealed an additional ‘Slowly declining’ trajectory, comprising 6% of participants (see Figures S14 and S15). Moreover, the results of this study may not be generalizable to other occupational groups than municipal employees, as they were the only sample included. In addition, we acknowledge that some changes in the outcome could occur as a result of changes in the exposures. In our study, we included exposures only prior to retirement into the analyses, and thus, the analyses do not warrant the assessment of change in the exposures across time or after the individual has retired. Moreover, the use of self‐report measures may have led to socially desirable responding, as in all observational survey‐based research (van de Mortel 2008). In addition, the analyses of this study do not allow for causal inferences, and the trajectory memberships are only approximations based on probabilistic models.

We also identified several strengths of this study. By employing a person‐oriented approach, we were able to identify distinct, relatively homogeneous groups of retirees who are expected to follow similar patterns of developmental trajectories. This study further enhances our understanding of statutory retirement: specifically, the most prevalent form of retirement, which warrants additional investigation due to its widespread impact. Utilizing five survey phases allowed for a follow‐up period of up to 15 years, one of the longest durations reported in the literature, and enabled us to robustly examine developmental trajectories.

Another strength is that the Helsinki Health Study has a large sample with high response rates and low attrition (Laaksonen et al. 2008). Differences in emotional well‐being scores and exposures between participants retained in the sample and those who dropped out after Phase 1 were minimal (see Table S8). The sample used for this study was also reasonably sized and exclusion of participants or missing data was not likely to drive the analyses (see Tables S9–S11), which improves the reliability and generalizability of our findings. The trajectories identified for those who had emotional well‐being data from all five phases were similar in shape as in the current study (see Figures S14 and S15). In addition, we utilized a well‐validated and reliable RAND‐36 questionnaire as the dependent variable, ensuring that our outcomes are both accurate and comparable to other studies in the field. Moreover, our findings can be considered at least partially generalizable to (comparable) employee populations in other high‐income countries with similar labor market and welfare structures; however, caution is warranted in all generalizations.

## 5 Conclusion

This study found that most participants maintained high emotional well‐being throughout statutory retirement, with no major contribution from retirement. A comparatively smaller group of individuals experienced lower emotional well‐being before statutory retirement and a gradual improvement after, or saw an increase until retirement, followed by a rapid decline. Unfavorable social and health‐related factors before retirement were associated with these smaller trajectory groups of emotional well‐being. For example, those with a history of a mentally very strenuous job may be particularly at risk of decline in emotional well‐being after statutory retirement, and with tailored interventions we could see whether change in work conditions contributes to better emotional well‐being. The findings also suggest that policymakers should consider different retirement trajectories when making decisions, as raising the retirement age could have different effects on emotional well‐being depending on individuals' experiences prior to statutory retirement.

## Author Contributions

**Emmi‐Susanna Katajapuu:** writing – original draft (lead); writing – review and editing (equal); visualization (lead). **Pauliina Saha:** writing – original draft (supporting); writing – review and editing (equal). **Aapo Hiilamo:** conceptualization (equal); methodology (equal); investigation (equal); writing – review and editing (equal); supervision (equal). **Jatta Valkonen:** conceptualization (equal); data curation (lead); investigation (equal); project administration (equal), writing – review and editing (equal); supervision (equal). **Tea Lallukka:** conceptualization (equal); funding acquisition (lead); investigation (equal); methodology (equal); project administration (equal); writing – review and editing (equal); supervision (equal).

## Funding

K.E.‐S, S.P., S.J. and L.T. are supported by the Research Council of Finland (Grant #330527).

## Ethics Statement

The Helsinki Health Study protocol was approved by the Ethics Committee of the Faculty of Medicine, University of Helsinki (initially 11/1998, updated later), and the City of Helsinki health and personnel authorities (initially 10/1999, updated later).

## Consent

The authors have nothing to report.

## Conflicts of Interest

The authors declare no conflicts of interest.

## Supporting information


**Data S1:** sjop70071‐sup‐0001‐Supinfo.docx.

## Data Availability

Due to strict General Data Protection Regulation (GDPR) laws, the Helsinki Health Study data cannot be publicly shared. The survey data are restricted to use by the research group for scientific purposes. For collaboration inquiries, please contact the principal investigators at kttl-hhs@helsinki.fi.
