# Supplementary material for: Emotional Well‐Being Trajectories Before and After Statutory Retirement—Contributions of Social and Health‐Related Factors
Source: Scand J Psychol. 2026 Jan 14;67(3):840–51. doi: 10.1111/sjop.70071 (PMC13159507; doi:10.1111/sjop.70071)

**SUPPLEMENTARY MATERIAL: TABLES**

**Supplementary Table 1**

*Selection Statistics of Emotional Well-being Trajectory Models of Statutory Retirees Including Posterior Probabilities of Trajectory Group Membership. The Helsinki Health Study 2000*–*2022 (N=5076, 81% Women)*

|  | **Trajectory model** | | | |
| --- | --- | --- | --- | --- |
|  | **Model 1** | **Model 2** | **Model 3** | **Model 4** |
| **Number of latent classes** | 1 | 2 | **3** | 4 |
| **Number of parameters** | 10 | 14 | **18** | 22 |
| **AIC** | 185371.2 | 184606.8 | **184230.9** | 184916.0 |
| **BIC** | 185436.5 | 184698.3 | **184348.5** | 185059.7 |
| **% group 1**  **(n)** | 100.0 | 87.4  (N=4436) | **85.0**  **(N=4314)** | 90.0  (N=4566) |
| **% group 2**  **(n)** |  | 12.6  (N=640) | **11.8**  **(N=601)** | 0.0  (N=0) |
| **% group 3**  **(n)** |  |  | **3.2**  **(N=161)** | 10.0  (N=510) |
| **% group 4**  **(n)** |  |  |  | 0.0  (N=0) |
| **Mean of posterior probabilities** |  |  |  |  |
| probability 1 | - | 0.97 | **0.95** | 0.43 |
| probability 2 | - | 0.86 | **0.84** | 0 |
| probability 3 | - | - | **0.77** | 0.65 |
| probability 4 | - | - | **-** | 0 |
| **Posterior probabilities above threshold > 0.7 (%)** |  |  |  |  |
| group 1 | - | 96.5 | **95.1** | 0 |
| group 2 | - | 78.6 | **75.5** | - |
| group 3 | - | - | **62.1** | 43.7 |
| group 4 | - | - | **-** | - |

*Note.* AIC, Akaike Information Criterion; BIC, Bayesian Information Criterion

**Supplementary Table 2**

*Descriptive Statistics of the Continuous Variables Among the Statutory Retiree Study Population, Stratified by Gender. The Helsinki Health Study 2000*–*2022 (N=5076, 81% Women)*

|  | **Mean (SD) (number of participants)** | | | | | |
| --- | --- | --- | --- | --- | --- | --- |
|  | **Women (N=4136)** | | **Men (N=940)** | | **Total (N=5076)** | |
|  | **Retired** | **Not retired** | **Retired** | **Not retired** | **Retired** | **Not retired** |
| **Age at Phase 1** | - | 52.8 (5.2) | - | 52.1 (5.1) | - | 52.3 (5.1) |
| **Emotional well-being** |  | | | | | |
| Phase 1 | - | 79.0 (16.4) (N=4110) | - | 78.7 (15.8) (N=934) | - | 78.7 (15.9) (N=5044) |
| Phase 2 | 82.6 (14.5) (N=779) | 78.9 (16.0) (N=3076) | 84.5 (13.3) (N=208) | 78.9 (16.5) (N=647) | 82.9 (14.4) (N=987) | 78.7 (16.1) (N=3723) |
| Phase 3 | 81.6 (15.0) (N=1800) | 79.7 (15.2) (N=1989) | 83.4 (14.5) (N=428) | 77.9 (17.1) (N=393) | 82.0 (14.9) (N=2228) | 79.4 (15.5) (N=2382) |
| Phase 4 | 80.8 (15.0) (N=2698) | 78.5 (15.8) (N=980) | 81.6 (15.7) (N=633) | 80.3 (15.9) (N=195) | 81.0 (15.1) (N=3331) | 78.8 (15.9) (N=1175) |
| Phase 5 | 78.0 (16.2) (N=3437) | - | 81.2 (15.8) (N=749) | - | 78.6 (16.2) (N=4186) | - |

*Note.* SD, standard deviation.

**Supplementary Table 3**

*Descriptive Statistics^a^ of the Categorical Variables Among the Statutory Retiree Study Population, Stratified by Gender, and p-values of Chi-Squared Tests. The Helsinki Health Study 2000*–*2022 (N=5076, 81% Women)*

|  | **Gender, n (%)** | |  |  |
| --- | --- | --- | --- | --- |
|  | **Women (N=4136)** | **Men (N=940)** | ***p*-value of Chi-Squared test** | **Total, n (%)** |
| **Parental education^b^** |  |  | <0.001 |  |
| Higher education | 521 (12.6) | 158 (16.8) |  | 679 (13.4) |
| Intermediate education | 378 (9.1) | 115 (12.2) |  | 493 (9.7) |
| Basic education | 3237 (78.3) | 667 (71.0) |  | 3904 (76.9) |
| **Marital status** |  |  | <0.001 |  |
| Married/cohabiting | 2834 (68.5) | 787 (83.7) |  | 3621 (71.3) |
| Other | 1302 (31.5) | 153 (16.3) |  | 1455 (28.7) |
| **Participant’s own education^b^** |  |  | <0.001 |  |
| Higher education | 1111 (26.9) | 360 (38.3) |  | 1471 (29.0) |
| Intermediate education | 1253 (30.3) | 248 (26.4) |  | 1501 (29.6) |
| Basic education | 1772 (42.8) | 332 (35.3) |  | 2104 (41.4) |
| **Physical workload^c^** |  |  | <0.001 |  |
| Physically non-strenuous (light) | 708 (17.1) | 330 (35.1) |  | 1038 (20.4) |
| Intermediate (rather light) | 1509 (36.5) | 371 (39.5) |  | 1880 (37.0) |
| Physically strenuous (rather strenuous/very strenuous) | 1346 (32.5) | 102 (10.9) |  | 1448 (28.5) |
| Missing | 573 (13.9) | 137 (14.6) |  | 710 (14.0) |
| **Mental workload^d^** |  |  | <0.001 |  |
| Mentally non-strenuous (light/very light) | 942 (22.8) | 291 (31.0) |  | 1233 (24.3) |
| Intermediate (rather strenuous) | 2162 (52.3) | 432 (46.0) |  | 2594 (51.1) |
| Mentally strenuous (very strenuous) | 435 (10.5) | 79 (8.4) |  | 514 (10.1) |
| Missing | 597 (14.4) | 138 (14.7) |  | 735 (14.5) |
| **LTPA^e^** |  |  | <0.001 |  |
| Vigorous activity | 376 (9.1) | 180 (19.1) |  | 556 (11.0) |
| Moderate activity | 2048 (49.5) | 394 (41.9) |  | 2442 (48.1) |
| Inactivity | 1712 (41.4) | 366 (38.9) |  | 2078 (40.9) |
| **Alcohol consumption^f^** |  |  | <0.001 |  |
| No binge-drinking | 3866 (93.5) | 730 (77.7) |  | 4596 (90.5) |
| Binge-drinking | 270 (6.5) | 210 (22.3) |  | 480 (9.5) |
| **Smoking^g^** |  |  | 0.009 |  |
| No | 3579 (86.5) | 782 (83.2) |  | 4361 (85.9) |
| Yes | 557 (13.5) | 158 (16.8) |  | 715 (14.1) |
| **F&V^h^** |  |  | <0.001 |  |
| Daily consumption | 2524 (61.0) | 341 (36.3) |  | 2865 (56.4) |
| Non-daily consumption | 1612 (39.0) | 599 (63.7) |  | 2211 (43.6) |
| **BMI^i^** |  |  | <0.001 |  |
| Normal weight | 1826 (44.1) | 338 (36.0) |  | 2164 (42.6) |
| Overweight | 1503 (36.3) | 420 (44.7) |  | 1923 (37.9) |
| Obesity | 807 (19.5) | 182 (19.4) |  | 989 (19.5) |
| **Sleep problems^j^** |  |  | <0.001 |  |
| No sleep problems | 396 (9.6) | 135 (14.4) |  | 531 (10.5) |
| Occasional sleep problems | 2638 (63.8) | 598 (63.6) |  | 3236 (63.8) |
| Frequent sleep problems | 1102 (26.6) | 207 (22.0) |  | 1309 (25.8) |
| **Mental disorder diagnosis^k^** |  |  | <0.001 |  |
| No | 2982 (72.1) | 772 (82.1) |  | 3754 (74.0) |
| Yes | 719 (17.4) | 99 (10.5) |  | 818 (16.1) |
| Missing | 435 (10.5) | 69 (7.3) |  | 504 (9.9) |

*Note.* LTPA, leisure-time physical activity; F&V, fruit and vegetable consumption; BMI, body mass index

^a^ Age, gender, parental education level, marital status, and participant’s education level measured at Phase 1. Other covariates measured at the phase before participant’s retirement.

^b^ Basic education = primary or secondary school or less; Intermediate education = matriculation or equivalent; Higher education = university degree or more

^c^ Physically non-strenuous = very light; Intermediate = rather light; Physically strenuous = rather strenuous/very strenuous

^d^ Mentally non-strenuous = very light/rather light; Intermediate = rather strenuous; Mentally strenuous = very strenuous

^e^ Vigorous activity = ≥ 14 MET-hours/week including the two highest intensity grades; Moderate activity = ≥ 14 MET-hours/week including the two lowest intensity grades; Inactivity = < 14 MET-hours/week

^f^ No binge-drinking = consuming six or more units of alcohol in a single instance once a month or less often; Binge-drinking = consuming six or more units of alcohol in a single instance once a week or more often

^g^ No = currently not a regular smoker; Yes = currently a regular smoker

^h^ Daily consumption = daily consumption of fruits and vegetables; Non-daily consumption = consuming fruits and vegetables less than daily

^i^ Normal weight = BMI < 25 kg/m^2^; Overweight = 25.0 ≤ BMI < 30.0 kg/m^2^; Obesity = BMI ≥ 30.0 kg/m^2^

^j^ No sleeping problems = no sleeping problems in the past four weeks; Occasional sleeping problems = any sleeping problems during 14 nights per four weeks or less; Frequent sleeping problems = any symptoms during more than 14 nights per four weeks

^k^ No = no previously diagnosed mental health diagnoses received from a doctor; Yes = a previously diagnosed mental health diagnoses received from a doctor

**Supplementary Table 4**

*Descriptive Statistics of the Continuous Variables Among the Statutory Retiree Study Population, Stratified by Emotional Well-being Trajectory Group Memberships. The Helsinki Health Study 2000*–*2022 (N=5076, 81% Women)*

|  | **Trajectory groups, mean (SD) (number of participants)** | | | | | |  | |
| --- | --- | --- | --- | --- | --- | --- | --- | --- |
|  | **Group 1 – Stable high (N=4314)** | | **Group 2 – Slowly increasing (N=601)** | | **Group 3 – Fast increasing, then fast decreasing (N=161)** | | **Total (N=5076)** | |
|  | **Retired** | **Not retired** | **Retired** | **Not retired** | **Retired** | **Not retired** | **Retired** | **Not retired** |
| **Age at Phase 1** | - | 52.2 (5.1) | - | 51.9 (5.0) |  | 54.1 (4.4) | - | 52.3 (5.1) |
| **Emotional well-being** |  | | | | | | | |
| Phase 1 | - | 82.1 (12.4) (N=4289) | - | 63.1 (17.7) (N=595) | - | 46.9 (21.6) (N=160) | - | 78.7 (15.9) (N=5044) |
| Phase 2 | 86.1 (10.8) (N=845) | 82.2 (12.9) (N=3176) | 57.3 (14.7) (N=100) | 57.2 (17.7) (N=452) | 81.4 (12.4) (N=42) | 70.0 (17.6) (N=95) | 82.9 (14.3) (N=987) | 78.9 (16.0) (N=3723) |
| Phase 3 | 85.5 (10.6) (N=1901) | 82.8 (12.1) (n =2049) | 54.0 (15.6) (n =237) | 54.8 (16.1) (n =278) | 80.0 (12.2) (n =90) | 75.2 (14.3) (n =55) | 82.0 (14.9) (N=2228) | 79.4 (15.5) (N=2382) |
| Phase 4 | 84.5 (11.3) (N=2829) | 82.9 (11.3) (N=1006) | 55.6 (15.1) (N=367) | 52.8 (16.0) (N=155) | 74.9 (17.2) (N=135) | 76.9 (21.8) (N=14) | 81.0 (15.1) (N=3331) | 78.8 (15.9) (N=1175) |
| Phase 5 | 82.8 (11.8) (N=3553) | - | 54.1 (16.1) (N=489) | - | 58.6 (21.8) (N=144) | - | 78.6 (16.2) (N=4186) | - |

*Note.* SD, standard deviation.

**Supplementary Table 5**

*Descriptive Statistics^a^ of the Categorical Variables Among the Statutory Retiree Study Population, Stratified by Emotional Well-being Trajectory Group Memberships, and p-values of Chi-Squared Tests. The Helsinki Health Study 2000*–*2022 (N=5076, 81% Women)*

|  | **Trajectory groups, n (%)** | | | ***p*-value of Chi-Squared** | **Total, n (%)** |
| --- | --- | --- | --- | --- | --- |
|  | **Group 1 – Stable high (n = 4314)** | **Group 2 – Slowly increasing (n = 601)** | **Group 3 – Fast increasing, then fast decreasing (n = 161)** |  |  |
| **Gender** |  |  |  | 0.780 |  |
| Women | 3509 (81.3) | 496 (82.5) | 131 (81.4) |  | 4136 (81.5) |
| Men | 805 (18.7) | 105 (17.5) | 30 (18.6) |  | 940 (18.5) |
| **Parental education^b^** |  |  |  | 0.015 |  |
| Higher education | 553 (12.8) | 96 (16.0) | 30 (18.6) |  | 679 (13.4) |
| Intermediate education | 407 (9.4) | 68 (11.3) | 18 (11.2) |  | 493 (9.7) |
| Basic education | 3354 (77.7) | 437 (72.7) | 113 (70.2) |  | 3904 (76.9) |
| **Marital status** |  |  |  | < 0.001 |  |
| Married/cohabiting | 3125 (72.4) | 385 (64.1) | 111 (68.9) |  | 3621 (71.3) |
| Other | 1189 (27.6) | 216 (35.9) | 50 (31.1) |  | 1455 (28.7) |
| **Participant’s own education^b^** |  |  |  | 0.842 |  |
| Higher education | 1252 (29.0) | 170 (28.3) | 49 (30.4) |  | 1471 (29.0) |
| Intermediate education | 1286 (29.8) | 171 (28.5) | 44 (27.3) |  | 1501 (29.6) |
| Basic education | 1776 (41.2) | 260 (43.3) | 68 (42.2) |  | 2104 (41.4) |
| **Physical workload^c^** |  |  |  | < 0.001 |  |
| Physically non-strenuous (light) | 900 (20.9) | 102 (17.0) | 36 (22.4) |  | 1038 (20.4) |
| Intermediate (rather light) | 1628 (37.7) | 206 (34.3) | 46 (28.6) |  | 1880 (37.0) |
| Physically strenuous (rather strenuous/very strenuous) | 1229 (28.5) | 173 (28.8) | 46 (28.6) |  | 1448 (28.5) |
| Missing | 557 (12.9) | 120 (20.0) | 33 (20.5) |  | 710 (14.0) |
| **Mental workload^d^** |  |  |  | < 0.001 |  |
| Mentally non-strenuous (light/rather light) | 1124 (26.1) | 91 (15.1) | 18 (11.2) |  | 2594 (51.1) |
| Intermediate (rather light) | 2224 (51.6) | 286 (47.6) | 84 (52.2) |  | 514 (10.1) |
| Mentally strenuous (very strenuous) | 390 (9.0) | 98 (16.3) | 26 (16.1) |  | 735 (14.5) |
| Missing | 576 (13.4) | 126 (21.0) | 33 (20.5) |  | 2594 (51.1) |
| **LTPA^e^** |  |  |  | < 0.001 |  |
| Vigorous activity | 484 (11.2) | 56 (9.3) | 16 (9.9) |  | 556 (11.0) |
| Moderate activity | 2119 (49.1) | 253 (42.1) | 70 (43.5) |  | 2442 (48.1) |
| Inactivity | 1711 (39.7) | 292 (48.6) | 75 (46.6) |  | 2078 (40.9) |
| **Alcohol consumption^f^** |  |  |  | < 0.001 |  |
| No binge-drinking | 3960 (91.8) | 505 (84.0) | 131 (81.4) |  | 4596 (90.5) |
| Binge-drinking | 354 (8.2) | 96 (16.0) | 30 (18.6) |  | 480 (9.5) |
| **Smoking^g^** |  |  |  | < 0.001 |  |
| No | 3779 (87.6) | 456 (75.9) | 126 (78.3) |  | 4361 (85.9) |
| Yes | 535 (12.4) | 145 (24.1) | 35 (21.7) |  | 715 (14.1) |
| **F&V^h^** |  |  |  | < 0.001 |  |
| Daily consumption | 2525 (58.5) | 270 (44.9) | 70 (43.5) |  | 2865 (56.4) |
| Non-daily consumption | 1789 (41.5) | 331 (55.1) | 91 (56.5) |  | 2211 (43.6) |
| **BMI^i^** |  |  |  | 0.192 |  |
| Normal weight | 1867 (43.3) | 232 (38.6) | 65 (40.4) |  | 2164 (42.6) |
| Overweight | 1622 (37.6) | 242 (40.3) | 59 (36.6) |  | 1923 (37.9) |
| Obesity | 825 (19.1) | 127 (21.1) | 37 (23.0) |  | 989 (19.5) |
| **Sleep problems^j^** |  |  |  | < 0.001 |  |
| No sleep problems | 496 (11.5) | 21 (3.5) | 14 (8.7) |  | 531 (10.5) |
| Occasional sleep problems | 2880 (66.8) | 270 (44.9) | 86 (53.4) |  | 3236 (63.8) |
| Frequent sleep problems | 938 (21.7) | 310 (51.6) | 61 (37.9) |  | 1309 (25.8) |
| **Mental disorder diagnoses^k^** |  |  |  | < 0.001 |  |
| No | 3366 (78.0) | 310 (51.6) | 78 (48.4) |  | 3754 (74.0) |
| Yes | 527 (12.2) | 229 (38.1) | 62 (38.5) |  | 818 (16.1) |
| Missing | 421 (9.8) | 62 (10.3) | 21 (13.0) |  | 504 (9.9) |

*Note.* LTPA, leisure-time physical activity; F&V, fruit and vegetable consumption; BMI, body mass index

^a^ Age, gender, Parental education level, marital status, and participant’s education level measured at Phase 1. Other covariates measured at the phase before participant’s retirement

^b^ Basic education = primary or secondary school or less; Intermediate education = matriculation or equivalent; Higher education = university degree or more

^c^ Physically non-strenuous = very light; Intermediate = rather light; Physically strenuous = rather strenuous/very strenuous

^d^ Mentally non-strenuous = very light/rather light; Intermediate = rather strenuous; Mentally strenuous = very strenuous

^e^ Vigorous activity = ≥ 14 MET-hours/week including the two highest intensity grades; Moderate activity = ≥ 14 MET-hours/week including the two lowest intensity grades; Inactivity = < 14 MET-hours/week

^f^ No binge-drinking = consuming six or more units of alcohol in a single instance once a month or less often; Binge-drinking = consuming six or more units of alcohol in a single instance once a week or more often

^g^ No = currently not a regular smoker; Yes = currently a regular smoker

^h^ Daily consumption = daily consumption of fruits and vegetables; Non-daily consumption = consuming fruits and vegetables less than daily

^i^ Normal weight = BMI < 25 kg/m^2^; Overweight = 25.0 ≤ BMI < 30.0 kg/m^2^; Obesity = BMI ≥ 30.0 kg/m^2^

^j^ No sleeping problems = no sleeping problems in the past four weeks; Occasional sleeping problems = any sleeping problems during 14 nights per four weeks or less; Frequent sleeping problems = any symptoms during more than 14 nights per four weeks

^k^ No = no previously diagnosed mental health diagnoses received from a doctor; Yes = a previously diagnosed mental health diagnoses received from a doctor

**Supplementary Table 6**

*Multinomial Regression Results as Average Marginal Effects (AME) and Their 95% Confidence Intervals for the Statutory Retiree Study Population’s Emotional Well-Being Trajectories^a^. The Helsinki Health Study 2000*–*2022 (N=5076, 82% Women)*

|  | **Model 1^b^** | | | **Model 2^c^** | | | **Model 3^d^** | | |
| --- | --- | --- | --- | --- | --- | --- | --- | --- | --- |
|  | **Group 1 – Stable high** | **Group 2 – Slowly increasing** | **Group 3 – Fast increasing, then fast decreasing** | **Group 1 – Stable high** | **Group 2 – Slowly increasing** | **Group 3 – Fast increasing, then fast decreasing** | **Group 1 – Stable high** | **Group 2 – Slowly increasing** | **Group 3 – Fast increasing, then fast decreasing** |
|  | **AME [95% CI]** | | | **AME [95% CI]** | | | **AME [95% CI]** | | |
| **Age [mean per 10 years]** | -0.02 [-0.04, 0.01] | -0.02 [-0.03, 0.00] | 0.03 [0.01, 0.05] | -0.03 [-0.05, 0.00] | -0.02 [-0.03, 0.00] | 0.04 [0.02, 0.06] | -0.01 [-0.03, 0.01] | -0.02 [-0.03, 0.00] | 0.03 [0.01, 0.05] |
| **Gender [ref. Men]** |  |  |  |  |  |  |  |  |  |
| Women | -0.01 [-0.03, 0.02] | 0.01 [-0.02, 0.03] | 0.00 [-0.01, 0.01] | 0.00 [-0.03, 0.03] | 0.01 [-0.02, 0.03] | -0.01 [-0.02, 0.01] | -0.01 [-0.03, 0.02] | 0.01 [-0.02, 0.03] | 0.00 [-0.01, 0.02] |
| **Parental education [ref. Higher education]** |  |  |  |  |  |  |  |  |  |
| Intermediate education | 0.01 [-0.03, 0.05] | -0.01 [-0.05, 0.04] | -0.01 [-0.03, 0.02] | 0.02 [-0.03, 0.06] | 0.00 [-0.04, 0.04] | -0.03 [-0.04, 0.01] | 0.00 [-0.04, 0.05] | 0.00 [-0.04, 0.04] | -0.01 [-0.03, 0.02] |
| Basic education | 0.05 [0.01, 0.08] | -0.03 [-0.06, 0.00] | -0.02 [-0.03, 0.00] | 0.04 [0.01, 0.08] | -0.03 [-0.06, 0.01] | -0.02 [-0.04, 0.00] | 0.03 [0.00, 0.06] | -0.02 [-0.05, 0.01] | -0.01 [-0.03, 0.00] |
| **Marital status [ref. Married/cohabiting]** |  |  |  |  |  |  |  |  |  |
| Other | -0.05 [-0.07, -0.02] | 0.04 [0.02, 0.06] | 0.00 [-0.01, 0.01] | -0.04 [-0.06, -0.01] | 0.03 [0.01, 0.06] | 0.00 [-0.01, 0.01] | -0.02 [-0.05, 0.00] | 0.03 [0.01, 0.05] | 0.00 [-0.01, 0.01] |
| **Participant education [ref. Higher education]** |  |  |  |  |  |  |  |  |  |
| Intermediate education | 0.01 [-0.02, 0.03] | 0.00 [-0.03, 0.02] | 0.00 [-0.02, 0.01] | -0.01 [-0.03, 0.02] | 0.01 [-0.02, 0.03] | 0.00 [-0.03, 0.01] | 0.00 [-0.03, 0.03] | 0.00 [-0.02, 0.03] | 0.00 [-0.02, 0.01] |
| Basic education | -0.01 [-0.03, 0.02] | 0.01 [-0.01, 0.03] | 0.00 [-0.01, 0.01] | -0.03 [-0.06, 0.00] | 0.02 [0.00, 0.05] | 0.01 [-0.01, 0.02] | -0.01 [-0.03, 0.02] | 0.01 [-0.01, 0.03] | 0.00 [-0.02, 0.01] |
| **Physical workload [ref. Non-strenuous]** |  |  |  |  |  |  |  |  |  |
| Intermediate | 0.00 [-0.03, 0.03] | 0.01 [-0.01, 0.03] | -0.01 [-0.03, 0.00] | 0.01 [-0.02, 0.04] | 0.00 [-0.02, 0.03] | -0.01 [-0.03, 0.00] | -0.01 [-0.03, 0.02] | 0.02 [-0.01, 0.04] | -0.01 [-0.02, 0.00] |
| Very strenuous | -0.01 [-0.04, 0.01] | 0.02 [-0.01, 0.03] | -0.01 [-0.03, 0.00] | 0.01 [-0.03, 0.04] | 0.00 [-0.03, 0.03] | -0.01 [-0.03, 0.01] | -0.01 [-0.04, 0.01] | 0.02 [-0.01, 0.05] | -0.01 [-0.02, 0.01] |
| **Mental workload [ref. Non-strenuous]** |  |  |  |  |  |  |  |  |  |
| Intermediate | -0.05 [-0.08, -0.03] | 0.04 [0.02, 0.06] | 0.02 [0.01, 0.03] | -0.06 [-0.08, -0.04] | 0.04 [0.02, 0.06] | 0.02 [0.01, 0.03] | -0.05 [-0.07, 0.03] | 0.03 [0.01, 0.05] | 0.02 [0.01, 0.03] |
| Very strenuous | -0.15 [-0.19, -0.11] | 0.12 [0.08, 0.15] | 0.04 [0.02, 0.06] | -0.15 [-0.20, - 0.11] | 0.12 [0.08, 0.16] | 0.04 [0.02, 0.06] | -0.10 [-0.13, -0.06] | 0.07 [0.04, 0.10] | 0.03 [0.01, 0.05] |
| **LTPA [ref. Vigorous activity]** |  |  |  |  |  |  |  |  |  |
| Moderate activity | -0.03 [-0.05, 0.02] | 0.01 [-0.02, 0.03] | 0.01 [0.00, 0.03] | -0.04 [-0.07, -0.01] | 0.02 [-0.01, 0.05] | 0.02 [0.00, 0.03] | 0.02 [-0.02, 0.06] | -0.03 [-0.06, 0.01] | 0.01 [-0.01, 0.03] |
| Inactivity | -0.08 [-0.11, -0.05] | 0.07 [0.04, 0.09] | 0.01 [0.00, 0.03] | -0.09 [-0.12, -0.06] | 0.08 [0.05, 0.11] | 0.01 [-0.01, 0.02] | -0.02 [-0.05, 0.02] | 0.01 [-0.03, 0.05] | 0.01 [-0.01, 0.02] |
| **Alcohol consumption [ref. No binge drinking]** |  |  |  |  |  |  |  |  |  |
| Binge-drinking | -0.11 [-0.16, -0.07] | 0.09 [0.05, 0.13] | 0.02 [0.00, 0.05] | -0.09 [-0.14, ­-0.05] | 0.07 [0.03, 0.11] | 0.02 [0.00, 0.04] | -0.04 [-0.08, -0.01] | 0.03 [0.00, 0.06] | 0.01 [-0.01, 0.03] |
| **Smoking [ref. No]** |  |  |  |  |  |  |  |  |  |
| Yes | -0.12 [-0.16, -0.08] | 0.10 [0.07, 0.13] | 0.02 [0.01, 0.04] | -0.11 [-0.15, -0.07] | 0.09 [0.05, 0.12] | 0.02 [0.00, 0.04] | -0.07 [-0.11, -0.04] | 0.06 [0.03, 0.09] | 0.02 [0.00, 0.03] |
| **F&V [ref. Daily consumption]** |  |  |  |  |  |  |  |  |  |
| Non-daily consumption | -0.07 [-0.09, - 0.05] | 0.06 [0.04, 0.08] | 0.01 [0.00, 0.02] | -0.07 [-0.10, -0.05] | 0.06 [0.04, 0.08] | 0.02 [0.00, 0.03] | -0.04 [-0.07, -0.02] | 0.03 [0.02, 0.05] | 0.01 [0.00, 0.02] |
| **BMI [ref. Normal weight]** |  |  |  |  |  |  |  |  |  |
| Overweight | -0.02 [-0.04, 0.00] | 0.02 [0.00, 0.04] | 0.00 [-0.01, 0.01] | -0.02 [-0.04, 0.01] | 0.01 [-0.01, 0.04] | 0.00 [-0.01, 0.01] | -0.01 [-0.03, 0.02] | 0.00 [-0.02, 0.02] | 0.00 [-0.01, 0.01] |
| Obesity | -0.03 [-0.06, 0.00] | 0.02 [-0.01, 0.04] | 0.01 [0.00, 0.03] | -0.03 [-0.06, 0.00] | 0.02 [-0.01, 0.04] | 0.01 [0.00, 0.03] | 0.01 [-0.02, 0.03] | -0.02 [-0.04, 0.01] | 0.01 [0.00, 0.03] |
| **Sleep problems [ref. No sleep problems]** |  |  |  |  |  |  |  |  |  |
| Occasional problems | -0.03 [-0.05, -0.01] | 0.02 [0.00, 0.05] | 0.01 [-0.01, 0.02] | -0.05 [-0.08, -0.03] | 0.05 [0.03, 0.07] | 0.00 [-0.01, 0.02] | -0.03 [-0.05, 0.00] | 0.02 [0.00, 0.05] | 0.00 [-0.01, 0.02] |
| Frequent problems | -0.18 [-0.22, -0.15] | 0.17 [0.14, 0.20] | 0.01 [0.00, 0.03] | -0.19 [-0.22, -0.15] | 0.18 [0.15, 0.21] | 0.01 [-0.01, 0.03] | -0.15 [-0.19, -0.12] | 0.15 [0.12, 0.18] | 0.01 [-0.01, 0.02] |
| **Mental disorder diagnoses [ref. No]** |  |  |  |  |  |  |  |  |  |
| Yes | -0.26 [-0.29, -0.22] | 0.20 [0.17, 0.23] | 0.05 [0.04, 0.07] | -0.23 [-0.27, -0.19] | 0.18 [0.15, 0.22] | 0.05 [0.03, 0.07] | -0.22 [-0.25, -0.18] | 0.16 [0.13, 0.19] | 0.05 [0.03, 0.07] |

*Note*. AME, average marginal effect; CI, confidence interval; LTPA, leisure-time physical activity; F&V, fruit and vegetable consumption; BMI, body mass index

^a^ Age, gender, parental education level, marital status, and participant’s education level measured at Phase 1. Other covariates measured at the phase before participant’s retirement

^b^ Adjusted for age and gender

^c^ Adjusted for age, gender, parental education, marital status, participant’s education, physical and mental strenuousness of work

^d^ Adjusted for age, gender, LTPA, alcohol consumption, smoking, F&V, BMI, sleep problems, and mental health diagnoses

**Supplementary Table 7**

*Multinomial Regression Results as Odds Ratios With 95% Confidence Intervals and Group 1 (“Stable High”) as the Reference Group for the Statutory Retiree Study Population’s (N = 5076) Emotional Well-Being Trajectories^a^. The Helsinki Health Study 2000*–*2022 (N=5076, 81% Women)*

|  | **Model 1^b^** | | **Model 2^c^** | | **Model 3^d^** | |
| --- | --- | --- | --- | --- | --- | --- |
|  | **Group 2 – Slowly increasing** | **Group 3 – Fast increasing, then fast decreasing** | **Group 2 – Slowly increasing** | **Group 3 – Fast increasing, then fast decreasing** | **Group 2 – Fast increasing, then fast decreasing** | **Group 3 – Slowly increasing** |
|  | **OR [95% CI]** | | **OR [95% CI]** | | **OR [95% CI]** | |
| **Age [mean per 10 years]** | 0.86 [0.73, 1.02] | 2.08 [1.51, 2.88] | 0.89 [0.74, 1.08] | 2.47 [1.70, 3.60] | 0.89 [0.74, 1.08] | 2.47 [1.70, 3.60] |
| **Gender [ref. Men]** | 1 | 1 | 1 | 1 | 1 | 1 |
| Women | 1.07 [0.86, 1.34] | 1.05 [0.70, 1.58] | 1.08 [0.82, 1.42] | 0.86 [0.54, 1.38] | 1.08 [0.82, 1.42] | 0.86 [0.54, 1.38] |
| **Parental education [ref. Higher education]** | 1 | 1 | 1 | 1 | 1 | 1 |
| Intermediate education | 0.96 [0.68, 1.34] | 0.85 [0.47, 1.55] | 0.97 [0.66, 1.43] | 0.60 [0.29, 1.24] | 1.02 [0.70, 1.48] | 0.83 [0.43, 1.62] |
| Basic education | 0.74 [0.59, 0.95] | 0.63 [0.42, 0.95] | 0.76 [0.57, 1.01] | 0.58 [0.36, 0.94] | 0.79 [0.60, 1.04] | 0.63 [0.40, 1.00] |
| **Marital status [ref. Married/cohabiting]** | 1 | 1 | 1 | 1 | 1 | 1 |
| Other | 1.48 [1.23, 1.77] | 1.16 [0.82, 1.64] | 1.40 [1.14, 1.73] | 1.22 [0.83, 1.80] | 1.31 [1.07, 1.61] | 0.98 [0.67, 1.43] |
| **Participant’s own education [ref. Higher education]** | 1 | 1 | 1 | 1 | 1 | 1 |
| Intermediate education | 0.96 [0.76, 1.20] | 0.94 [0.62, 1.42] | 1.08 [0.84, 1.41] | 0.94 [0.58, 1.53] | 1.03 [0.80, 1.32] | 0.93 [0.59, 1.45] |
| Basic education | 1.08 [0.88, 1.33] | 0.94 [0.64, 1.37] | 1.27 [0.97, 1.67] | 1.21 [0.74, 1.98] | 1.12 [0.88, 1.42] | 0.91 [0.60, 1.40] |
| **Physical workload [ref. Non-strenuous]** | 1 | 1 | 1 | 1 | 1 | 1 |
| Intermediate | 1.10 [0.86, 1.42] | 0.67 [0.43, 1.05] | 1.01 [0.78, 1.31] | 0.62 [0.39, 0.98] | 1.21 [0.91, 1.61] | 0.72 [0.44, 1.17] |
| Very strenuous | 1.20 [0.92, 1.57] | 0.91 [0.57, 1.45] | 1.01 [0.75, 1.35] | 0.78 [0.47, 1.31] | 1.26 [0.93, 1.70] | 0.85 [0.51, 1.41] |
| **Mental workload [ref. Non-strenuous]** | 1 | 1 | 1 | 1 | 1 | 1 |
| Intermediate | 1.57 [1.23, 2.01] | 2.39 [1.42, 4.00] | 1.61 [1.25, 2.07] | 2.50 [1.48, 4.21] | 1.57 [1.19, 2.06] | 2.29 [1.31, 3.99] |
| Very strenuous | 3.07 [2.25, 4.17] | 4.16 [2.25, 7.70] | 3.14 [2.28, 4.32] | 4.45 [2.31, 8.18] | 2.30 [1.62, 3.27] | 3.52 [1.80, 6.88] |
| **LTPA [ref. Vigorous activity]** | 1 | 1 | 1 | 1 | 1 | 1 |
| Moderate activity | 1.11 [0.81, 1.50] | 1.59 [0.84, 3.01] | 1.37 [0.92, 2.05] | 2.01 [0.91, 4.44] | 1.37 [0.92, 2.05] | 2.01 [0.91, 4.44] |
| Inactivity | 1.89 [1.39, 2.57] | 1.85 [0.96, 3.55] | 2.37 [1.58, 3.54] | 2.11 [0.93, 4.76] | 2.37 [1.58, 3.54] | 2.11 [0.93, 4.76] |
| **Alcohol consumption [ref. No binge drinking]** | 1 | 1 | 1 | 1 | 1 | 1 |
| Binge-drinking | 2.08 [1.60, 2.70] | 2.08 [1.28, 3.36] | 1.93 [1.43, 2.60] | 2.03 [1.19, 3.45] | 1.93 [1.43, 2.60] | 2.03 [1.19, 3.45] |
| **Smoking [ref. No]** | 1 | 1 | 1 | 1 | 1 | 1 |
| Yes | 2.24 [1.80, 2.80] | 1.98 [1.30, 3.00] | 2.21 [1.71, 2.85] | 2.03 [1.27, 3.25] | 2.21 [1.71, 2.85] | 2.03 [1.27, 3.25] |
| **F&V [ref. Daily consumption]** | 1 | 1 | 1 | 1 | 1 | 1 |
| Non-daily consumption | 1.74 [1.46, 2.07] | 1.70 [1.23, 2.34] | 1.86 [1.52, 2.27] | 1.84 [1.28, 2.66] | 1.86 [1.52, 2.27] | 1.84 [1.28, 2.66] |
| **BMI [ref. Normal weight]** | 1 | 1 | 1 | 1 | 1 | 1 |
| Overweight | 1.19 [0.98, 1.44] | 1.16 [0.81, 1.68] | 1.16 [0.93, 1.45] | 1.15 [0.76, 1.74] | 1.16 [0.93, 1.45] | 1.15 [0.76, 1.74] |
| Obesity | 1.20 [0.95, 1.51] | 1.52 [1.01, 2.28] | 1.21 [0.93, 1.58] | 1.64 [1.03, 2.60] | 1.21 [0.93, 1.58] | 1.64 [1.03, 2.60] |
| **Sleep problems [ref. No sleep problems]** | 1 | 1 | 1 | 1 | 1 | 1 |
| Occasional problems | 1.44 [1.02, 2.04] | 1.25 [0.73, 2.14] | 2.59 [1.52, 4.42] | 1.24 [0.67, 2.31] | 2.59 [1.52, 4.42] | 1.24 [0.67, 2.31] |
| Frequent problems | 4.71 [3.32, 6.69] | 1.86 [1.04, 3.33] | 7.97 [4.65, 13.66] | 1.64 [0.83, 3.22] | 7.97 [4.65, 13.66] | 1.64 [0.83, 3.22] |
| **Mental disorder diagnoses [ref. No]** | 1 | 1 | 1 | 1 | 1 | 1 |
| Yes | 4.80 [3.95, 5.83] | 5.03 [3.54, 7.13] | 4.62 [3.70, 5.77] | 4.62 [3.08, 6.92] | 4.62 [3.70, 5.77] | 4.62 [3.08, 6.92] |

*Note*. OR, odds ratio; CI, confidence interval; LTPA, leisure-time physical activity; F&V, fruit and vegetable consumption; BMI, body mass index

^a^ Age, gender, parental education level, marital status, and participant’s education level measured at Phase 1. Other covariates measured at the phase before participant’s retirement

^b^ Adjusted for age and gender

^c^ Adjusted for age, gender, parental education, marital status, participant’s education, physical and mental strenuousness of work

^d^ Adjusted for age, gender, LTPA, alcohol consumption, smoking, F&V, BMI, sleep problems, and mental health diagnoses

**Supplementary Table 8**

*Comparison of Key Variables Between Participants Who Dropped Out After Phase 1 and Those Included in the Final Sample. The Helsinki Health Study 2000*–*2022 (81% Women)*

| **Variable** | **Dropped out after Phase 1 (*N* = 513)** | **Included in the analysis (*N* = 5076)** |
| --- | --- | --- |
| **Gender** | Men = 156  Women = 357 | Men = 940  Women = 4136 |
| **Mean age at Phase 1** | 51.7 | 52.3 |
| **Mean of emotional well-being at Phase 1** | 77.0 | 78.7 |
| **Diagnosis** | No = 339  Yes = 106  NA = 68 | No = 3426  Yes = 818  NA = 832 |
| **Mental workload** | Non-strenuous = 145  Intermediately strenuous = 279  Strenuous = 69  NA = 20 | Non-strenuous = 1233  Intermediately strenuous = 2594  Strenuous = 514  NA = 735 |

**Supplementary Table 9**

*Number of Missing Values in Emotional Well-being Across Phases by Trajectory Group. The Helsinki Health Study 2000*–*2022 (N=5076, 81% Women)*

| **Trajectory group** | **Number of missing values** | | | | | | | | | |
| --- | --- | --- | --- | --- | --- | --- | --- | --- | --- | --- |
|  | **Phase 1** | | **Phase 2** | | **Phase 3** | | **Phase 4** | | **Phase 5** | |
|  | **N** | **%** | **N** | **%** | **N** | **%** | **N** | **%** | **N** | **%** |
| 1 | 25 | 0.6 | 11 | 0.3 | 12 | 0.3 | 138 | 3.2 | 68 | 1.6 |
| 2 | 6 | 1.0 | 0 | 0 | 6 | 1.0 | 22 | 3.7 | 7 | 1.1 |
| 3 | 1 | 0.6 | 2 | 1.2 | 1 | 0.6 | 6 | 3.7 | 0 | 0 |
| Total | 32 | 0.6 | 13 | 0.3 | 19 | 0.4 | 166 | 3.6 | 75 | 1.8 |

**Supplementary Table 10**

*Descriptive Statistics of Continuous Variables of Statutory Retirees Included in the Sample vs. Combined Included and Excluded^a^ from the Sample. The Helsinki Health Study 2000–2022 (N=5076, 81% Women)*

| **Variable** | **Included in the sample (N=5076) (mean, SD)** | **Combination of those included in and excluded from the sample (N=5511) (mean, SD)** |
| --- | --- | --- |
| **Age** | 52.3 (5.1) | 52.4 (5.1) |
| **Emotional well-being** | 78.7 (15.2) | 78.7 (16.1) |

^a^ Excluded from the sample refers to cases with less than three emotional well-being measurement points or who had missing or inconsistent information on timing of statutory retirement

**Supplementary Table 11**

*Descriptive Statistics of Categorical Variables of Statutory Retirees Included in the Sample vs. Combined Included and Excluded^a^ from the Sample. The Helsinki Health Study 2000–2022 (N=5076, 81% Women)*

| **Variable** | **Included in the sample (N=5076), %** | **Combination of those included in and excluded from the sample (N=5511), %** |
| --- | --- | --- |
| **Gender** |  |  |
| Men | 18.5 | 19.8 |
| Women | 81.5 | 80.2 |
| **Parental education** |  |  |
| Higher education | 13.4 | 13.2 |
| Intermediate education | 9.7 | 9.6 |
| Basic education | 76.9 | 77.2 |
| **Marital status** |  |  |
| Married/cohabiting | 71.3 | 71.0 |
| Other | 28.7 | 29.1 |
| **Own education** |  |  |
| Higher education | 29.0 | 28.3 |
| Intermediate education | 29.6 | 28.9 |
| Basic education | 41.5 | 42.8 |
| **Physical workload** |  |  |
| Physically non-strenuous (light) | 21.7 | 21.0 |
| Intermediate (rather light) | 43.6 | 43.6 |
| Physically strenuous (rather strenuous/very strenuous) | 33.2 | 33.7 |
| Missing | 1.5 | 1.7 |
| **Mental workload** |  |  |
| Mentally non-strenuous (light/rather light) | 22.6 | 22.9 |
| Intermediate (rather light) | 61.5 | 61.0 |
| Mentally strenuous (very strenuous) | 13.9 | 13.9 |
| Missing | 1.9 | 2.1 |
| **LTPA** |  |  |
| Vigorous activity | 11.9 | 11.1 |
| Moderate activity | 48.8 | 48.7 |
| Inactivity | 39.4 | 39.3 |
| **Alcohol consumption** |  |  |
| No binge drinking | 90.9 | 88.0 |
| Binge drinking | 9.1 | 9.7 |
| **Smoking** |  |  |
| No | 80.2 | 78.7 |
| Yes | 19.8 | 20.4 |
| **F&V** |  |  |
| Daily consumption | 51.5 | 50.5 |
| Non-daily consumption | 48.5 | 49.0 |
| **BMI** |  |  |
| Normal weight | 50.6 | 48.9 |
| Overweight | 35.3 | 35.7 |
| Obesity | 14.1 | 14.4 |
| **Sleep problems** |  |  |
| No sleep problems | 13.5 | 12.9 |
| Occasional sleep problems | 66.3 | 65.8 |
| Frequent sleep problems | 20.2 | 20.4 |
| **Mental disorder diagnoses** |  |  |
| No | 74.0 | 73.5 |
| Yes | 16.1 | 16.1 |
| Missing | 9.9 | 10.3 |

*Note.* M, mean; SD, standard deviation*;* LTPA, leisure-time physical activity; F&V, fruit and vegetable consumption; BMI, body mass index. All covariates use their first respective category in the table as their reference group

^a^ Excluded from the sample refers to cases with less than three emotional well-being measurement points or who had missing or inconsistent information on timing of statutory retirement

**SUPPLEMENTARY MATERIAL: FIGURES**

**Supplementary Figure 1**

*Flow Diagram of Statutory Retiree Participant Selection for The Current Study. The Helsinki Health Study 2000*–*2022 (N=5076, 81% Women)*

**Analytical sample:**

**N=5076**

Excluded

N=435:

Missing information on emotional well-being in two or more phases (n=258); inconsistent or missing information on the timing of retirement (n=177)

**Target population:** 40 to 60-year-old employees of the City of Helsinki, Finland (2000-2002)

N=13 344

Other than statutorily retired during the follow-up (e.g., had not retired yet, went on disability pension)

N=3449

Statutorily retired during the follow-up (2000-2022)

N=5511

Responders

N=8960 (67%)

**Supplementary Figure 2**

*Selection Statistics of Trajectory Models for the Statutory Retiree Study Population. The Helsinki Health Study 2000*–*2022 (N=5076, 81% Women)*


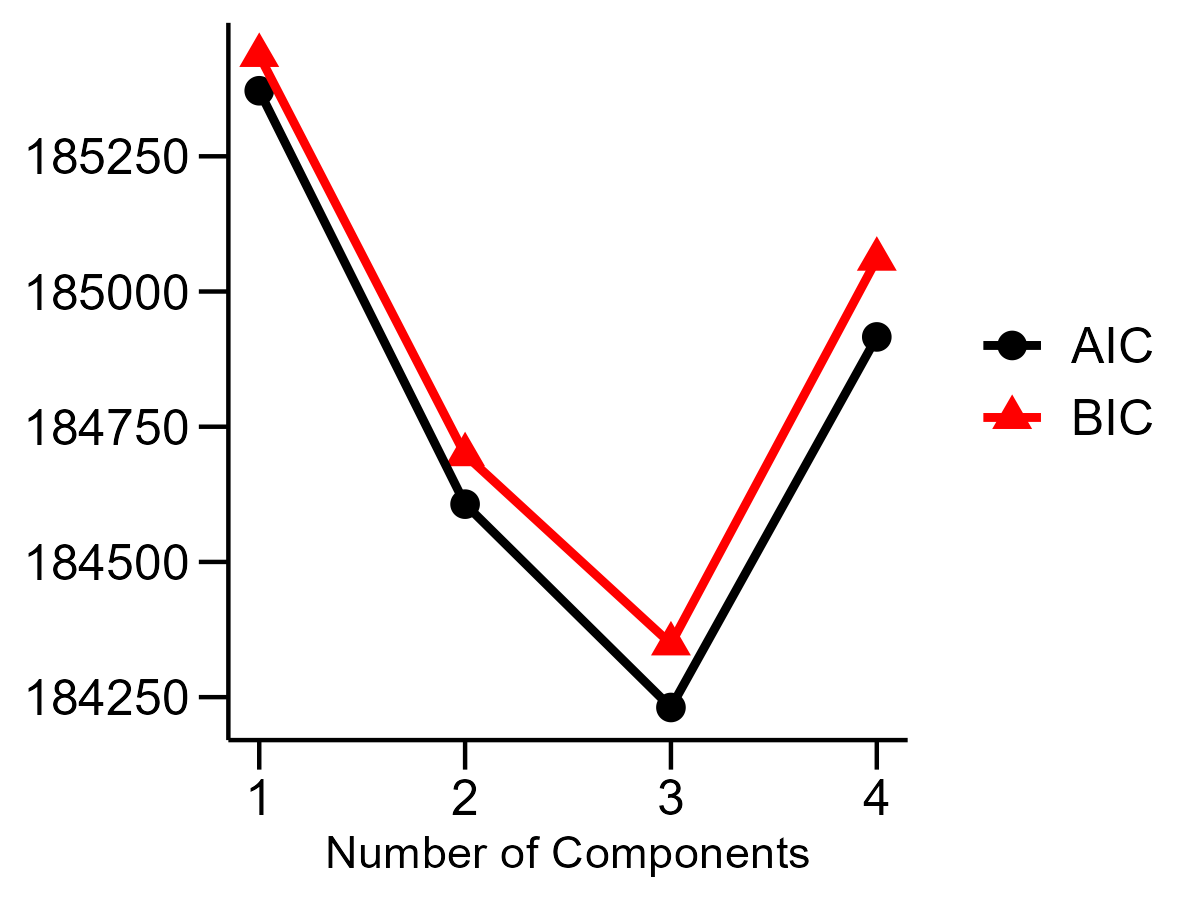


*Note.* AIC, Akaike Information Criterion; BIC, Bayesian Information Criterion

**Supplementary Figure 3**

*Posterior Probability Distributions of Trajectory Groups Among Statutory Retiree Participants. The Helsinki Health Study 2000*–*2022 (N=5076, 81% Women)*


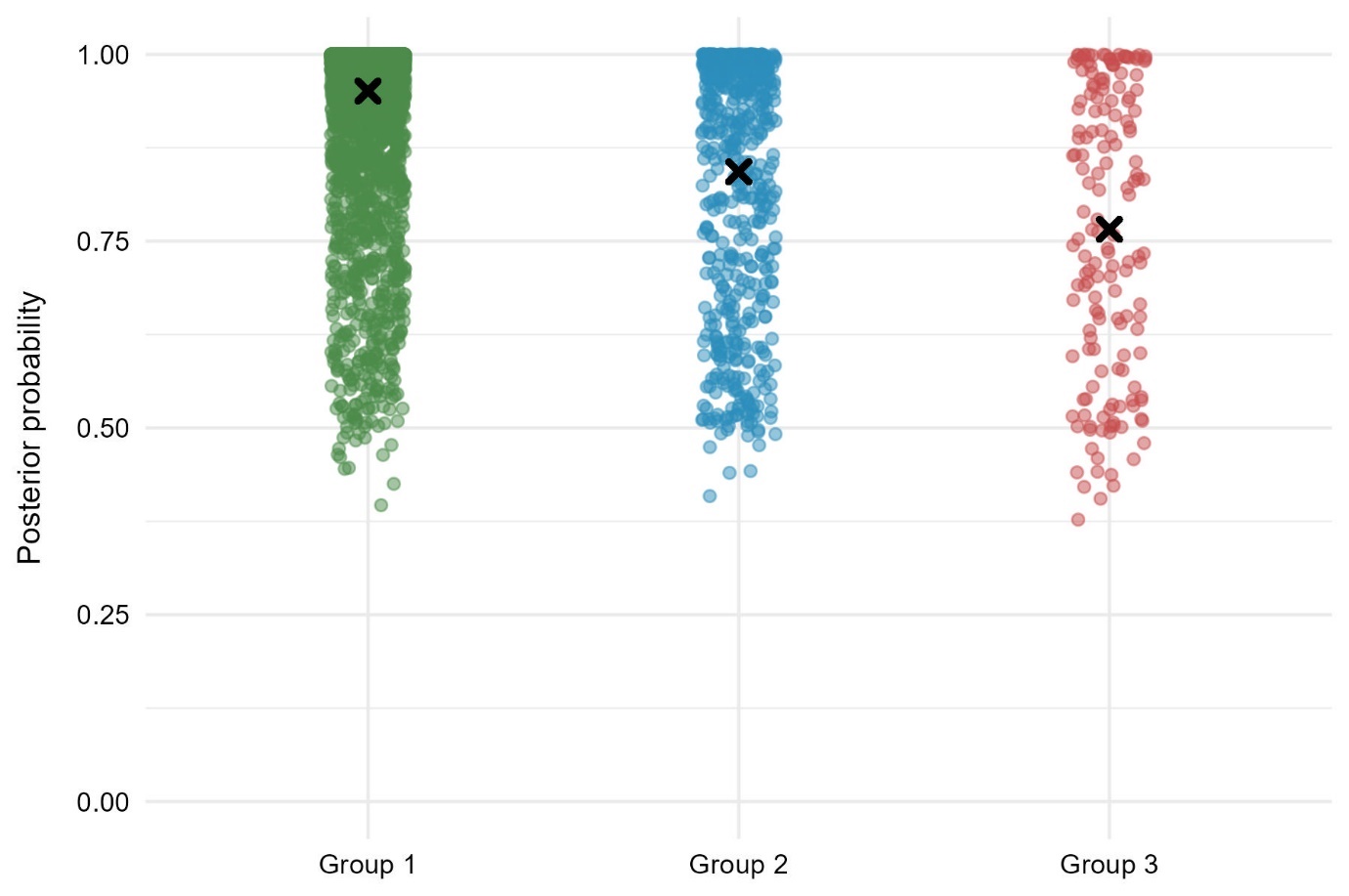


*Note.* Crosses indicate group means.

**Supplementary Figure 4**

*Cramer’s V Correlation Matrix of Social and Health-Related Factors Included in This Study. The Helsinki Health Study 2000*–*2022 (N=5076, 81% Women)*


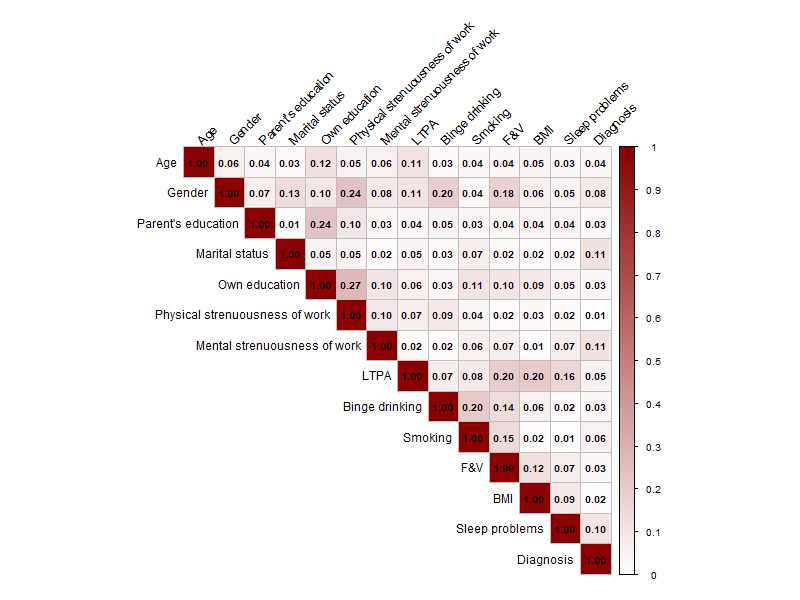


*Note.* LTPA, leisure-time physical activity; F&V, fruit and vegetable consumption; BMI, body mass index.

**Supplementary Figure 5**

*Count of Participants at Each Year of Time to Retirement, Stratified by Trajectory Group, to Show That Follow-Up Was Cut at 15 Years as There Are Not Many Emotional Well-Being Scores Past -15 and 15 Years. X-axis Shows Years Before and After Retirement With 0 Indicating the Retirement Year. The Helsinki Health Study 2000*–*2022 (N=5076, 81% Women)*


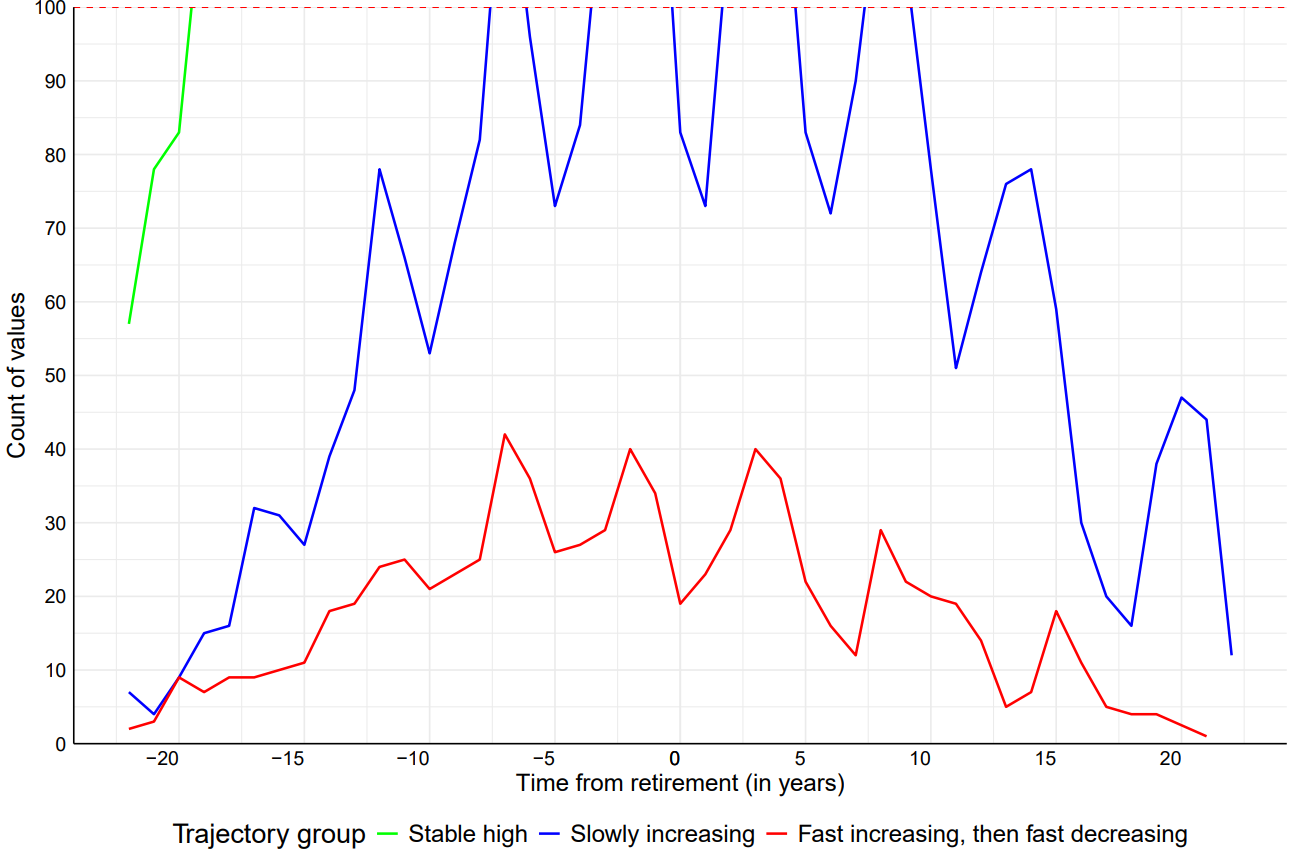


*Note.* Counts are truncated at 100 for simplicity and thus counts higher than 100 are not shown.

**Supplementary Figure 6**

*Spaghetti Plot Showing the Individual Emotional Well-being Trajectories Forming the ‘Stable High’ Trajectory Group (N = 4314; Thick Green Line). The Helsinki Health Study 2000*–*2022 (N=5076, 81% Women)*


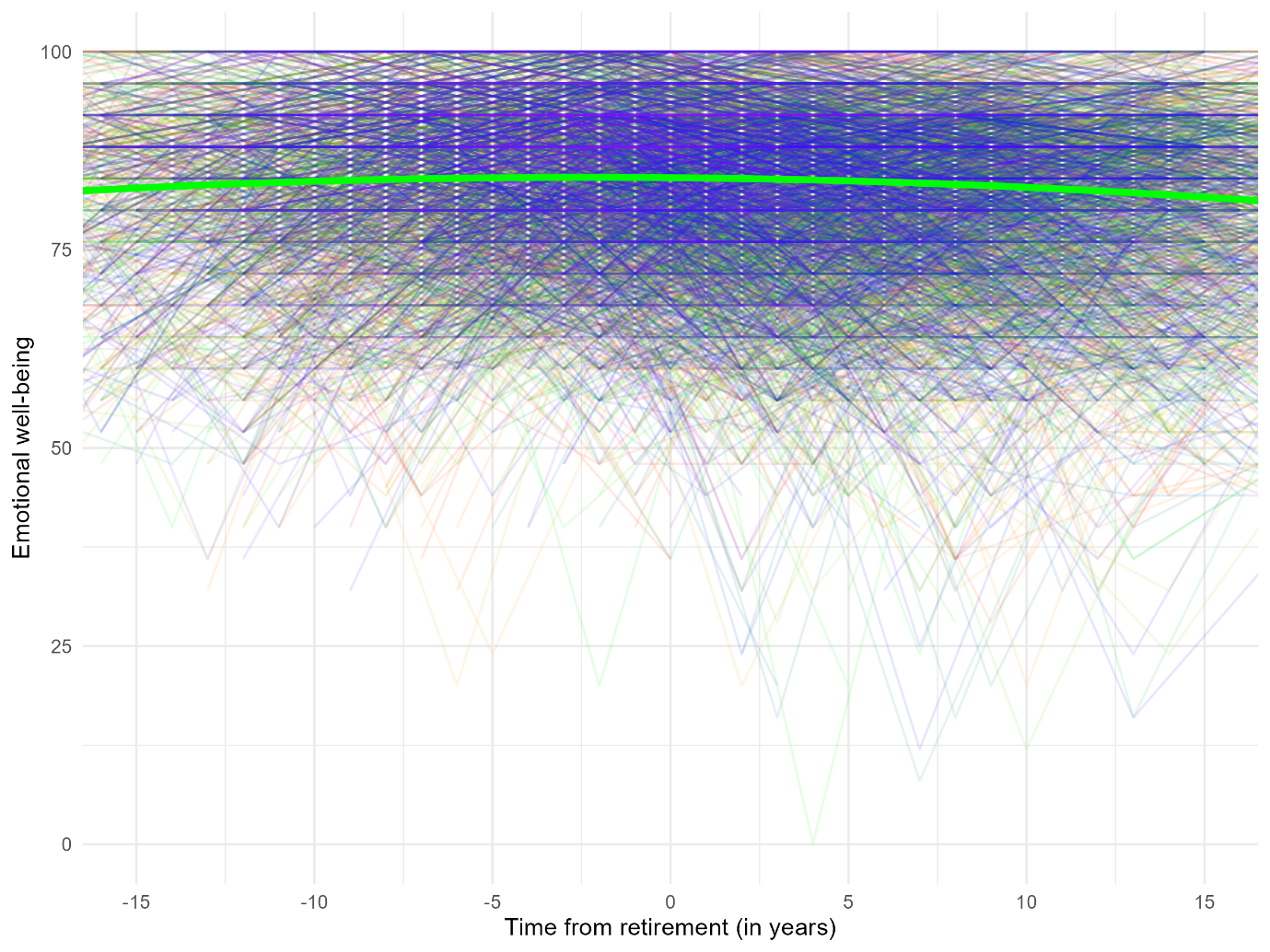


**Supplementary Figure 7**

*Spaghetti Plot Showing the Individual Emotional Well-being Trajectories Forming the ‘Slowly Increasing’ Trajectory Group (N = 601; Thick Blue Line). The Helsinki Health Study 2000*–*2022 (N=5076, 81% Women)*


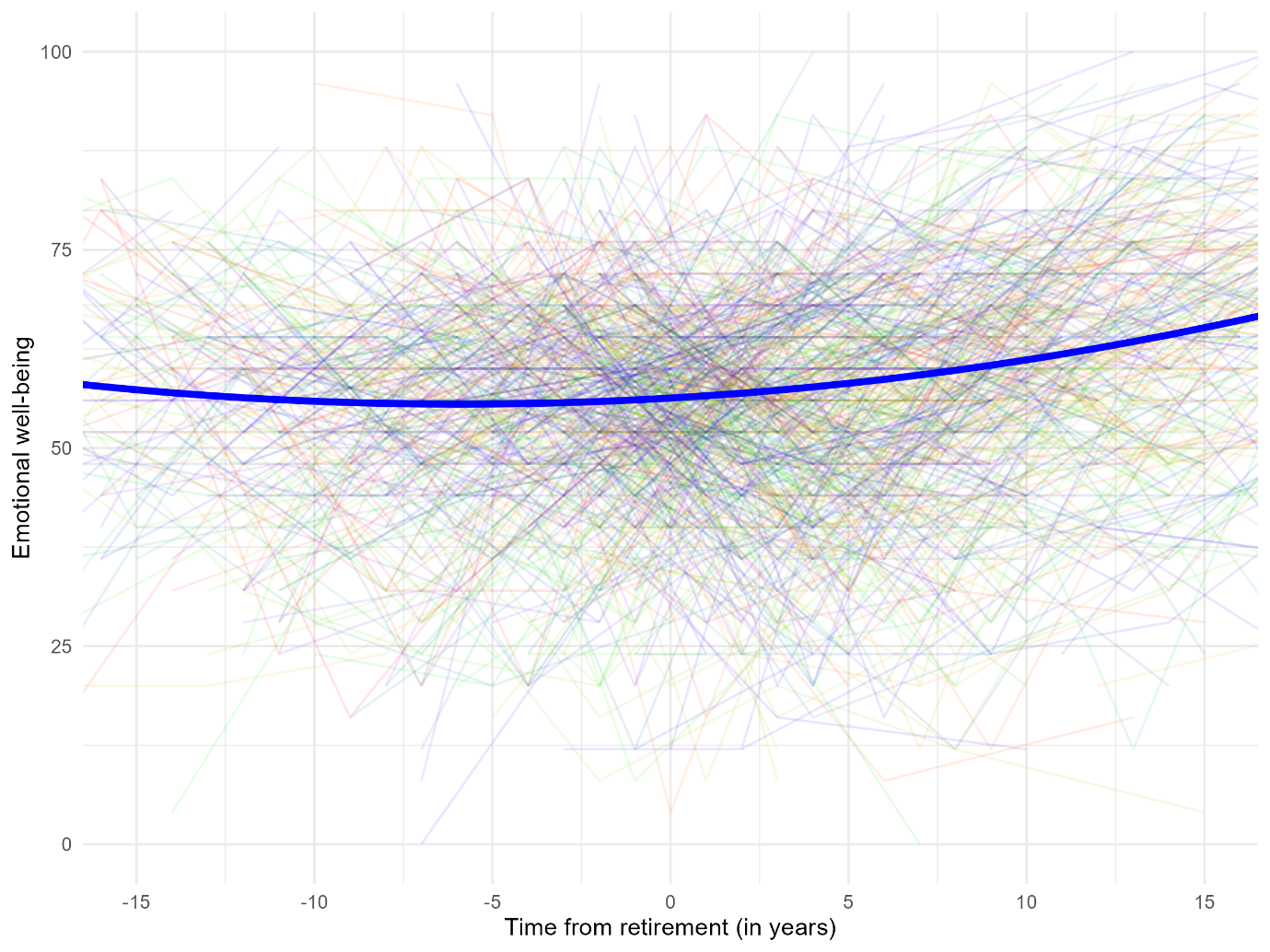


**Supplementary Figure 8**

*Spaghetti Plot Showing the Individual Emotional Well-being Trajectories Forming the ‘Fast Increasing, Then Fast Decreasing’ Trajectory Group (N = 161; Thick Red Line). The Helsinki Health Study 2000*–*2022 (N=5076, 81% Women)*


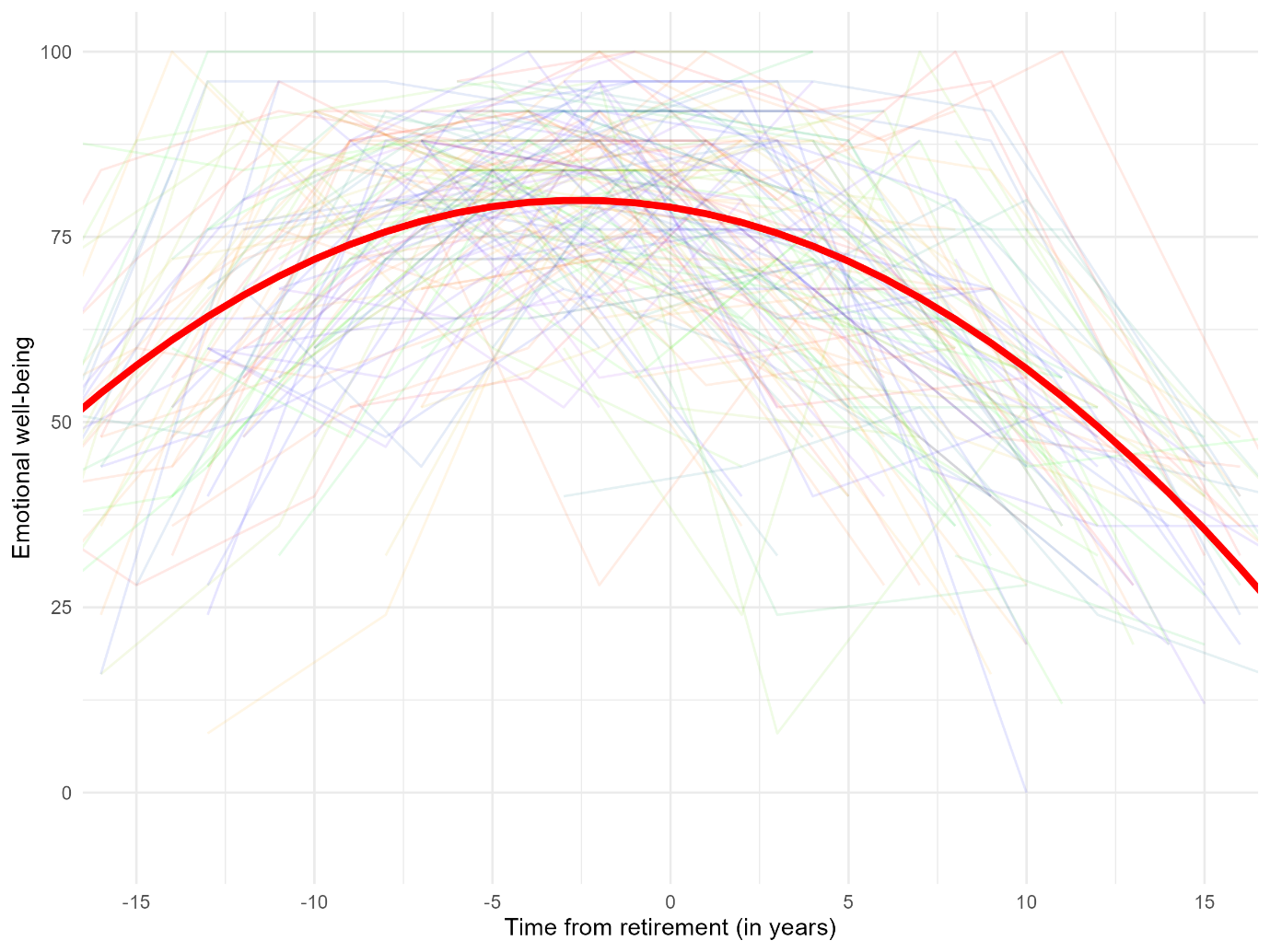


**Supplementary Figure 9**

*
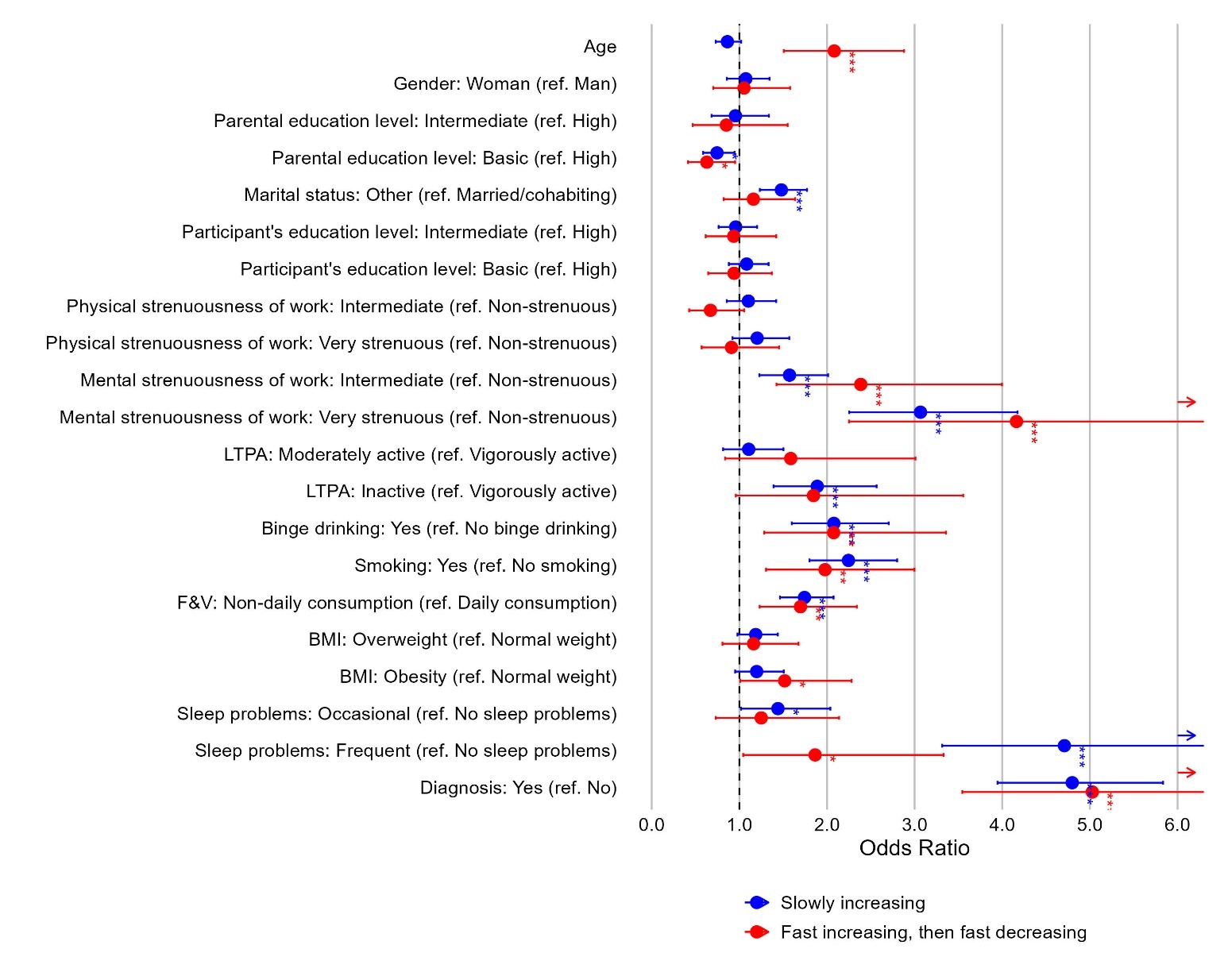
Multinomial Regression Results of Model 1 as Odds Ratios and Their 95% Confidence Intervals and Trajectory Group 1 (“Stable High”) as Reference for the Statutory Retiree Study Population’s Emotional Well-Being Trajectories. The Helsinki Health Study 2000*–*2022 (N=5076, 81% Women)*

*Note.* LTPA, leisure-time physical activity; F&V, fruit and vegetable consumption; BMI, body mass index. Age represented in increments of 10 years

**Supplementary Figure 10**

*Multinomial Regression Results of Model 2 as Average Marginal Effects (AME) and Their 95% Confidence Intervals for the Statutory Retiree Study Population’s Emotional Well-Being Trajectories. The Helsinki Health Study 2000*–*2022 (N=5076, 81% Women)*


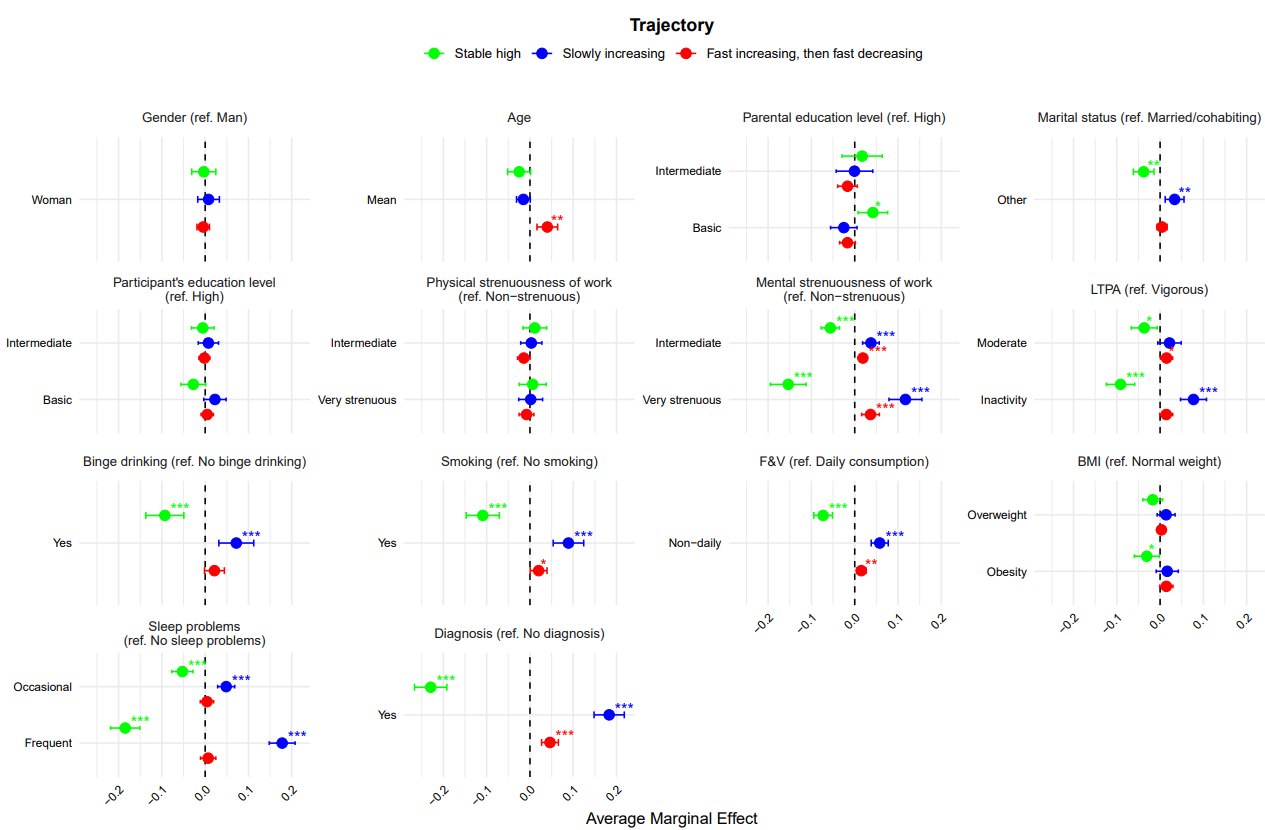


*Note.* LTPA, leisure-time physical activity; F&V, fruit and vegetable consumption; BMI, body mass index. Age represented in increments of 10 years

**Supplementary Figure 11**


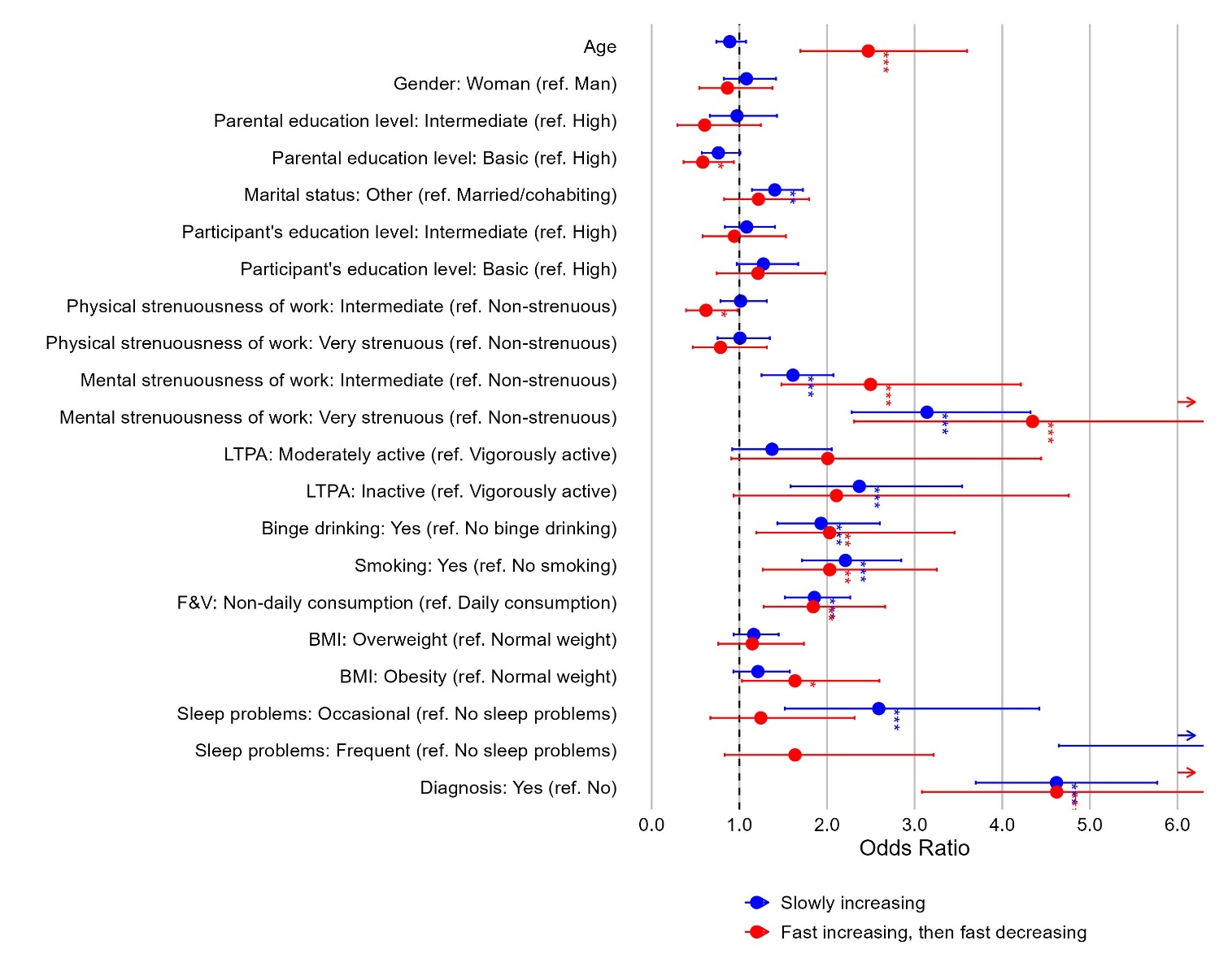
*Multinomial Regression Results of Model 2 as Odds Ratios and Their 95% Confidence Intervals and Trajectory Group 1 (“Stable High”) as Reference for the Statutory Retiree Study Population’s Emotional Well-Being Trajectories. The Helsinki Health Study 2000*–*2022 (N=5076, 81% Women)*

*Note.* LTPA, leisure-time physical activity; F&V, fruit and vegetable consumption; BMI, body mass index. Age represented in increments of 10 years

**Supplementary Figure 12**

*Multinomial Regression Results of Model 3 as Average Marginal Effects (AME) and Their 95% Confidence Intervals for the Statutory Retiree Study Population’s Emotional Well-Being Trajectories. The Helsinki Health Study 2000*–*2022 (N=5076, 81% Women)*


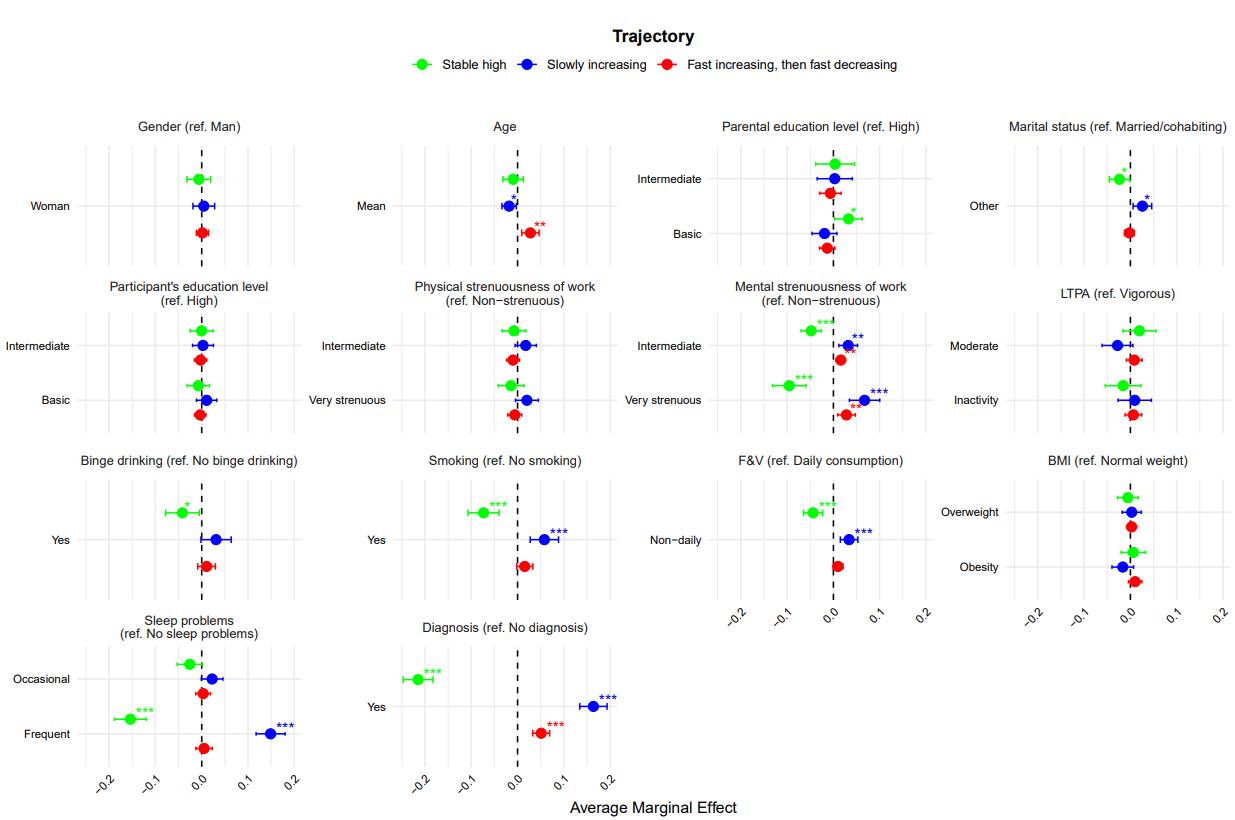


*Note.* LTPA, leisure-time physical activity; F&V, fruit and vegetable consumption; BMI, body mass index. Age represented in increments of 10 years

**Supplementary Figure 13**


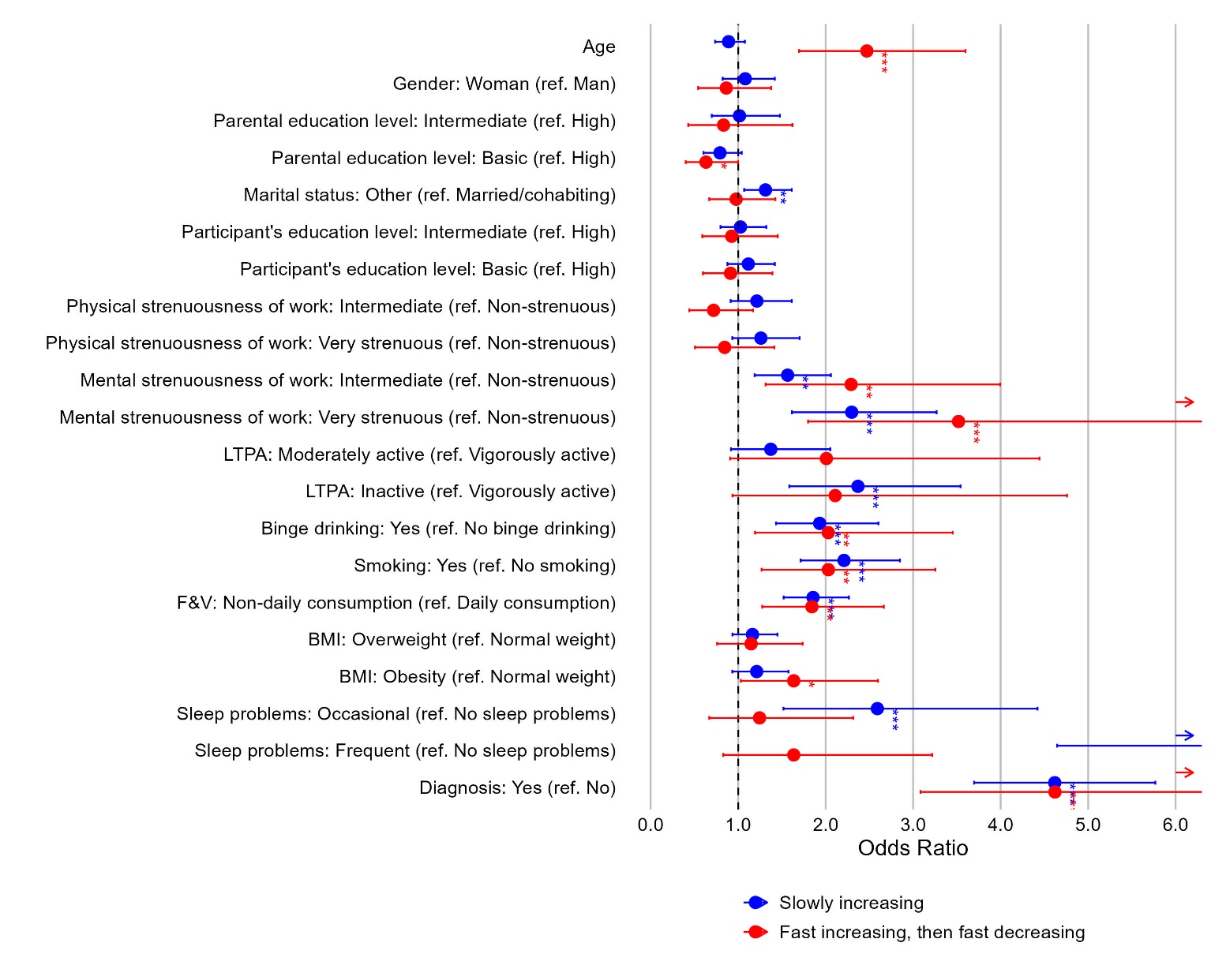
*Multinomial Regression Results of Model 3 as Odds Ratios and Their 95% Confidence Intervals and Trajectory Group 1 (“Stable High”) as Reference for the Statutory Retiree Study Population’s Emotional Well-Being Trajectories. The Helsinki Health Study 2000*–*2022 (N=5076, 81% Women)*

*Note.* LTPA, leisure-time physical activity; F&V, fruit and vegetable consumption; BMI, body mass index. Age represented in increments of 10 years

**Supplementary Figure 14**

*Emotional Well-being Trajectories of Those Who Had Emotional Well-being Data from All Five Phases (N=3308, 83% Women), Based on Different Class Solutions, Without Gender Interaction. Only Solutions of One and Two Classes Are Shown as Added Classes Always Had Zero Allocated Participants (so, N=0). Includes Group Means and Fitted Lines With 95% Confidence Intervals, Identified by Growth Mixture Modelling. Number of Participants in Each Group and the Prevalence of Group Sizes Are Shown. X-axis Shows Years Before and After Retirement With 0 Indicating the Retirement Year. The Helsinki Health Study 2000*–*2022 (N=5076, 81% Women)*


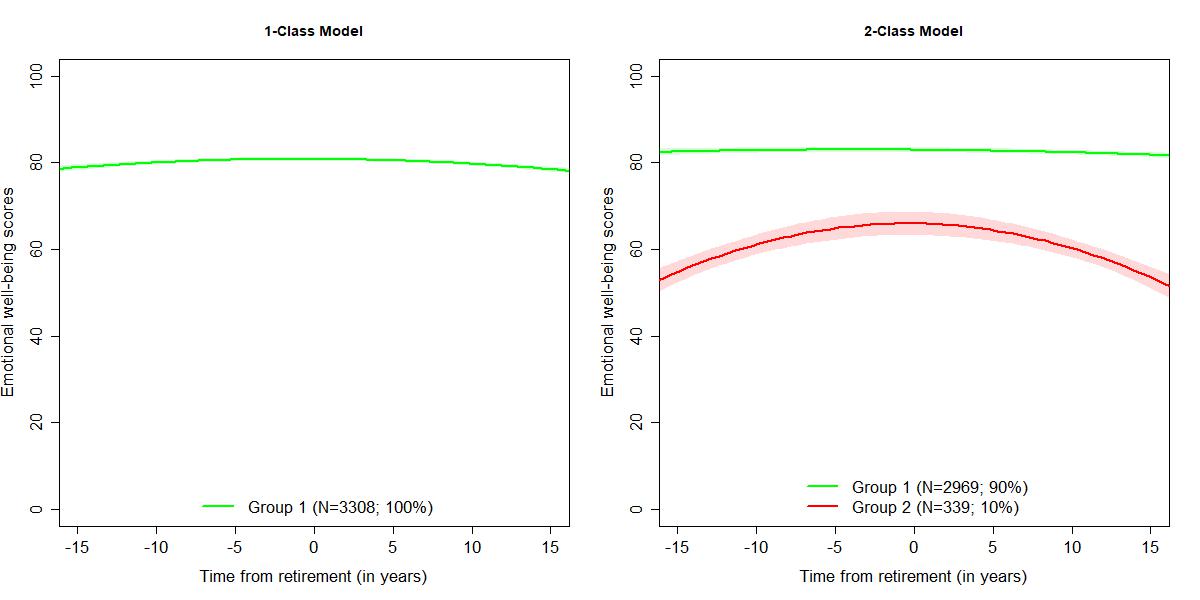


**Supplementary Figure 15**

*Emotional Well-being Trajectories of Those Who Had Emotional Well-being Data from All Five Phases (N=3308, 83% Women), Based on Different Class Solutions, With Gender Interaction. Includes Group Means and Fitted Lines With 95% Confidence Intervals, Identified by Growth Mixture Modelling. Number of Participants in Each Group and the Prevalence of Group Sizes Are Shown. X-axis Shows Years Before and After Retirement With 0 Indicating the Retirement Year. The Helsinki Health Study 2000*–*2022 (N=5076, 81% Women)*


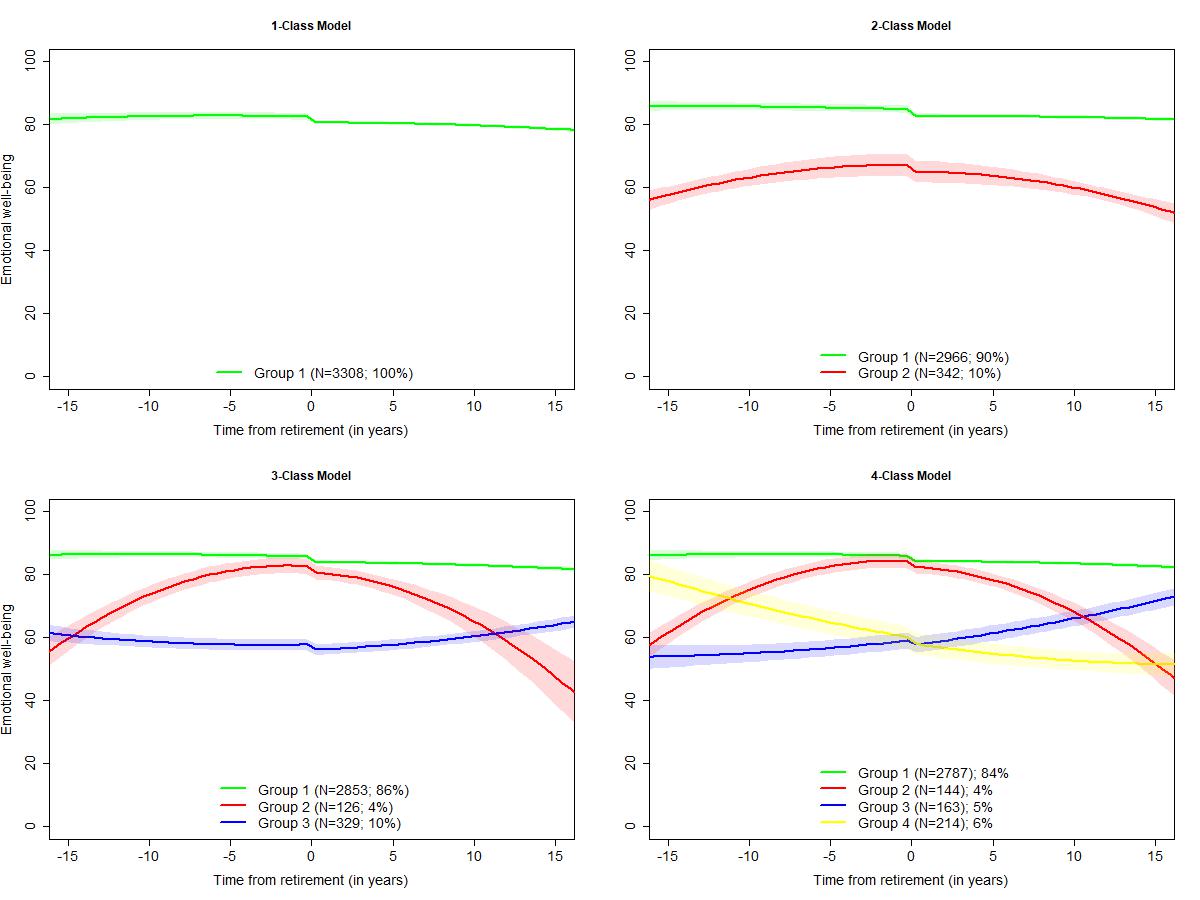

Supplement: Supplementary file 1 — Data S1: sjop70071‐sup‐0001‐Supinfo.docx. [file SJOP-67-840-s001.docx]
